# Revealing Internal Rotation and ^14^N Nuclear Quadrupole Coupling in the Atmospheric Pollutant 4-Methyl-2-nitrophenol: Interplay of Microwave Spectroscopy and Quantum Chemical Calculations

**DOI:** 10.3390/molecules28052153

**Published:** 2023-02-24

**Authors:** Shefali Baweja, Eleonore Antonelli, Safia Hussain, Antonio Fernández-Ramos, Isabelle Kleiner, Ha Vinh Lam Nguyen, M. Eugenia Sanz

**Affiliations:** 1 Department of Chemistry, King’s College London, Britannia House, 7 Trinity Street, London SE1 1DB, UK; 2 Université Paris Est Créteil and Université Paris Cité, CNRS, LISA, F-94010 Créteil, France; 3 Departamento de Química Física and Centro Singular de Investigación en Química Biolóxica e Materiais Moleculares (CIQUS), Jenaro de la Fuente s/n, Universidad de Santiago de Compostela, 15782 Santiago de Compostela, Spain; 4 Université Paris Cité and Université Paris Est Créteil, CNRS, LISA, F-75013 Paris, France; 5 Institut Universitaire de France (IUF), 1 rue Descartes, F-75231 Paris, France

**Keywords:** rotational spectroscopy, ab initio and density functional theory calculations, internal rotation, nuclear quadrupole coupling, large-amplitude motion

## Abstract

The structure and interactions of oxygenated aromatic molecules are of atmospheric interest due to their toxicity and as precursors of aerosols. Here, we present the analysis of 4-methyl-2-nitrophenol (4MNP) using chirped pulse and Fabry–Pérot Fourier transform microwave spectroscopy in combination with quantum chemical calculations. The rotational, centrifugal distortion, and ^14^N nuclear quadrupole coupling constants of the lowest-energy conformer of 4MNP were determined as well as the barrier to methyl internal rotation. The latter has a value of 106.4456(8) cm^−1^, significantly larger than those from related molecules with only one hydroxyl or nitro substituent in the same para or meta positions, respectively, as 4MNP. Our results serve as a basis to understand the interactions of 4MNP with atmospheric molecules and the influence of the electronic environment on methyl internal rotation barrier heights.

## 1. Introduction

**Figure 1:**
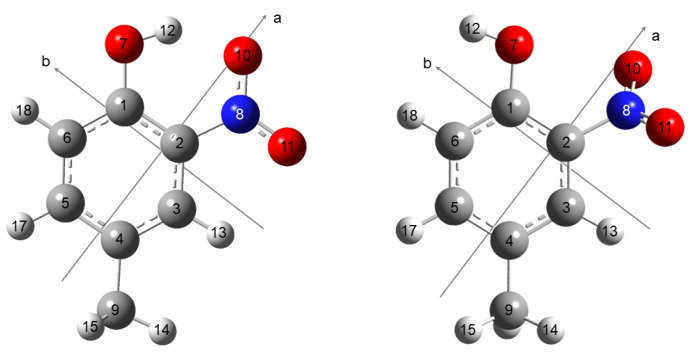
Molecular structures of the two conformers of 4-methyl-2-nitrophenol, 4MNP-1 (**left**) and 4MNP-2 (**right**). The carbon atoms are gray, hydrogen atoms white, oxygen atoms red, and the nitrogen atom is blue. The principal inertial axes *a*, *b* are indicated. The *c*-axis is perpendicular to the *ab* plane.

A major class of pollutants and aerosol precursors are oxygenated aromatic compounds, including functional groups such as –OH, –CO, and –NO_2_ [1,2,3,4]. They are primary products of combustion and have higher toxicity than their parent, non-oxygenated compounds [5]. Among the oxygenated aromatic molecules, phenols and nitrophenols have attracted much attention due to their toxicity [6]. These constitute a large portion of the volatile organic compounds (VOCs) in urban areas, and their subsequent reactions are pivotal in determining atmospheric chemistry because they increase the proportion of oxidants, such as OH radicals, and the formation of secondary organic aerosol [7,8]. *p*-Cresol is one of the main atmospheric phenols, directly released to the atmosphere as a byproduct of diesel combustion and wood burning, as well as produced from photochemical reactions of other aromatic molecules. Its oxidation with NO_3_ radicals produces 4-methyl-2-nitrophenol (4MNP, see Figure 1), whose photolysis was recently discovered to be a potential source of the OH radical in polluted suburban environments [7]. 4MNP is also an important component of “brown carbon” from biomass burning [9,10] and is released into the atmosphere by combustion [11,12]. Despite their relevance to the Earth’s atmosphere, there are fundamental gaps in our knowledge of the competing reaction pathways for these pollutants, their evolution from the first aggregation stages and cluster growth to aerosol formation, and their interactions with water [13,14].

To advance our understanding of the interactions of oxygenated aromatic compounds with other molecules in the atmosphere, it is necessary to characterize their structures and relative configurations. This can be achieved by applying high-resolution spectroscopic techniques in combination with high-level quantum chemistry calculations. Rotational spectroscopy [15,16] is a powerful technique for characterizing structures and large amplitude motions, such as methyl internal rotation, in the gas phase [17,18,19]. It can identify without ambiguity different conformers and isomers present in the sample because small differences in their structures will result in them showing different rotational spectra. Rotational spectroscopy has been successfully applied to investigate many atmospherically important molecules including amines, oxygenated organic compounds, terpenes, and their complexes [20,21,22,23,24,25,26]. Moreover, the results obtained from this experimental technique can be used to benchmark the performance of different theoretical methods.

In this study, we report the investigation of 4MNP, an aerosol precursor and pollutant [27,28,29,30,31,32], via a combination of rotational spectroscopy and quantum chemical calculations. 4MNP has a methyl group in para and meta with respect to the hydroxyl and nitro groups, respectively. The methyl torsion is expected to experience a *V*_3_ barrier, with each rotational transition split into two components, labeled A and E according to their symmetry. Only a few studies of methyl internal rotation barriers of doubly substituted toluene derivatives have been reported [17]. From the analysis of the rotational spectrum of 4MNP, we have determined its barrier to methyl internal rotation as well as its rotational and ^14^N nuclear quadrupole coupling constants.

## 2. Results

### 2.1. Computational

Geometry optimizations of 4MNP were initially performed with the Gaussian09 and Gaussian16 suites of programs [33,34] using density functional theory (DFT) [35,36] as well as ab initio second-order Møller–Plesset perturbation theory (MP2) [37] in combination with Pople’s 6-311G++(d,p) basis set [38,39]. The DFT calculations were carried out using the dispersion-corrected B3LYP-D3BJ functional, including Becke–Johnson damping [40,41] and the B3PW91 [36,42] functional, with tight optimization convergence criteria and an ultrafine grid. They yielded the computed rotational constants, dipole moment components, ^14^N nuclear quadrupole coupling constants (NQCCs), and energy differences between the conformers to guide the analysis of the experimental spectrum (see Table 1). Two conformers were obtained and are illustrated in Figure 1 with atomic numbering. Their atomic coordinates are available in Table S1 in the Supplementary Materials.

Because it has been reported that MP2 does not properly model the ^14^N nuclear quadrupole coupling of the –NO_2_ group [43,44], we also used two different DFT functionals to describe it. We chose B3LYP-D3BJ, as it usually provides a good description of structural parameters [21,45,46,47,48,49,50], and B3PW91, because it has been reported to be the best-performing method for describing nuclear quadrupole coupling [42]. Furthermore, we used Bailey’s method to compute the NQCCs by performing electric field gradient calculations at the B3PW91/6-311+G(d,p) level of theory on the molecular geometry optimized at the MP2/6-311++G(d,p) level [42]. The obtained values were corrected with the calibration factor eQ/h = −4.599 MHz a.u^−1^ recommended for molecules containing π-conjugation [51], yielding *χ_aa_* = −0.7053 MHz, *χ_bb_* = 0.0379 MHz, *χ_cc_* = 0.6674 MHz, and *χ_ab_* = 0.7078 MHz.

The methyl internal rotation barriers were calculated by rotating the methyl group in steps of 10° by varying the dihedral angle *α* = ∠(C3C4C9H14) and allowing the rest of the molecule to relax at each point. The height of the barrier is consistently larger for 4MNP-1 at all levels of theory. The obtained energy points were parameterized using a Fourier expansion with the coefficients collected in Table S2. Using these coefficients, the potential energy curves are drawn as contour plots in Figure S1. Calculations using the B3LYP-D3BJ and B3PW91 methods resulted in a potential curve with perfect threefold symmetry, which is typical for methyl internal rotation. At the MP2 level, we obtained a strange shape of the potential curve, where the minima are not located at 0°, 120°, and 240°, but are shifted by 10°.

**Figure 2:**
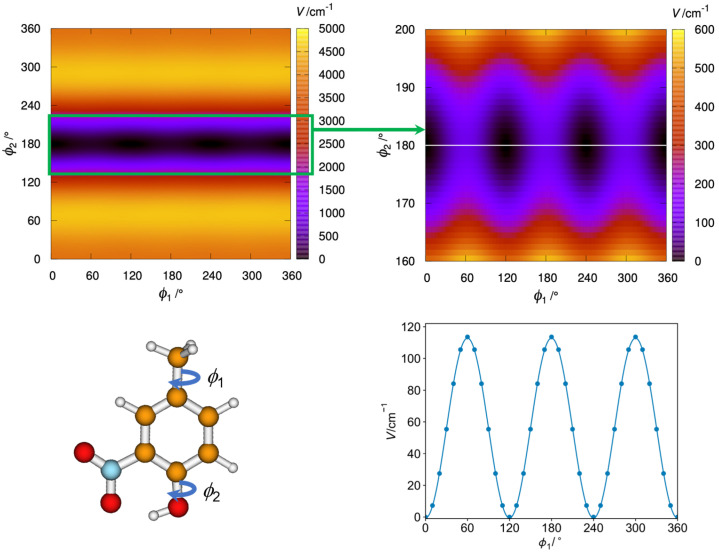
(**Top left**): Contour plot showing the variation of potential energy (in cm^−1^) with the internal rotation of the methyl $\left(\phi_{1}\right)$ and hydroxyl $\left(\phi_{2}\right)$ groups of 4MNP (**bottom left**). A zoom of the contour plot enhancing the region of the absolute minima is depicted in the (**top right**) position. A one-dimensional cut (white line in the zoomed contour plot) that passes through the equilibrium structures shows the fit of the *V*_3_ potential to the B3PW91 points (**bottom right**).

Furthermore, we carried out a two-dimensional contour plot using the B3PW91 method with the Q2DTor program [52] (see Figure 2). It shows the change in potential energy when the methyl and hydroxyl groups are rotated in steps of 10° while allowing the rest of the molecule to relax at each point. The resulting points were fitted to the Fourier series of Equation S1, which was employed to solve the 2D Schrödinger equation leading to the energy levels of Table S3 (see ref. [53] for details). There are three indistinguishable absolute minima corresponding to 4MNP-1 with C_s_ symmetry. The hydrogen atom of the –OH group points toward the –NO_2_, creating a strong hydrogen bond. This hydrogen bond in the 4MNP-1 structure avoids any internal rotation of the –OH group, and the molecule behaves as a system with a single methyl internal rotor. Notice that the comparison of the first 18th vibrational energy levels obtained from the diagonalization of the 1D and 2D potentials shows that these levels correspond exclusively to the vibrational excitation of the methyl group (see Table S3). 4MNP-2 lacks the hydrogen bond of 4MNP-1, and the repulsion between the oxygens of the –OH and –NO_2_ groups leads to a nonplanar structure with a very high energy above the global minimum. Therefore, the rotation of the hydroxyl group displaces the nitro group out of the phenylic plane, with angles of 31.1° (B3LYP-D3BJ), 31.0° (B3PW91), and 52.1° (MP2). Additionally, it affects the methyl group rotation, which presents a barrier of *ca*. 30 cm^−1^.

**Figure 3:**
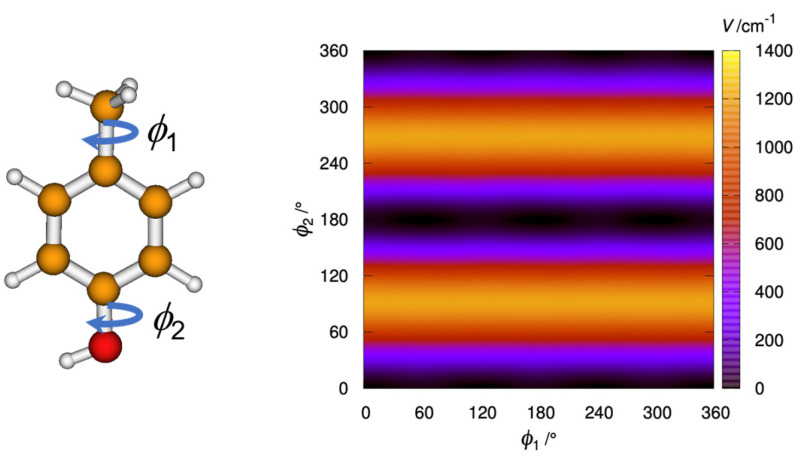
Contour plot showing the variation of potential energy (in cm^−1^) with the internal rotation of the methyl $\left(\phi_{1}\right)$ and hydroxyl $\left(\phi_{2}\right)$ groups of *p*-cresol, generated at the B3PWB91/6-311++G(d,p) level.

The effect of the –NO_2_ group is thus substantial. Comparing 4MNP with the related 4-methylphenol (*p*-cresol) shows that whereas 4MNP-2 is very high-energy, the analogous structure of *p*-cresol is the absolute minimum, and the analogous structure of 4MNP-1 is a transition state that is 17 cm^−1^ above the minimum. Therefore, due to the symmetry in *p*-cresol, there is only one minimum, with the two eclipsed hydrogen atoms of the rotating tops in *anti* configuration but with the torsion about the methyl group close to a free rotation (see Figure 3).

### 2.2. Rotational Spectrum

There are two possible conformers of 4MNP, depending on the orientation of the hydroxyl group (see Figure 1 and Table 1). The global minimum 4MNP-1 exhibits an intramolecular O–H∙∙∙O hydrogen bond between the hydrogen of the hydroxyl group and one of the oxygens of the nitro group. This stabilizing interaction is missing in conformer 4MNP-2, leading to a much higher relative energy. 4MNP-2 is thus not expected to be populated in our supersonic expansion.

**Figure 4:**
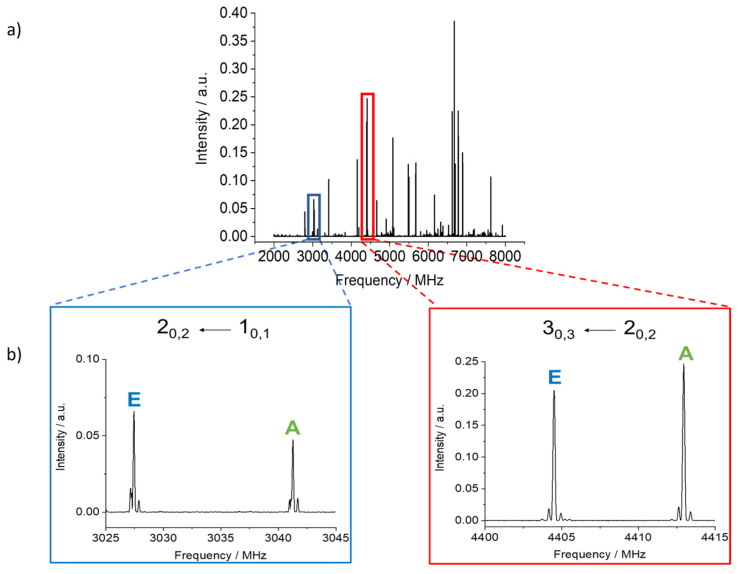
(**a**) The chirped pulse FTMW spectrum of 4MNP-1 recorded from 2 to 8 GHz. (**b**) Sections of the spectrum showing assigned transitions and torsional species.

**Figure 5:**
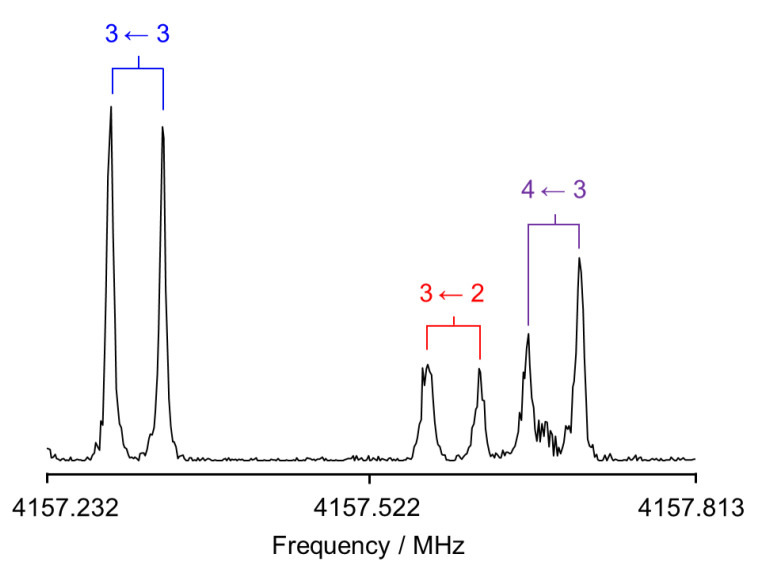
A typical resonator-based FTMW spectrum of the *a*-type transition 3_1,3_ ← 2_1,2_ showing its ^14^N nuclear quadrupole hyperfine structure. Each transition is split into two components due to the Doppler effect. The line was polarized at 4157.313 MHz, and 241 free induction decays were co-added.

4MNP-1 is predicted to be a planar molecule with only *µ_a_* and *µ_b_* dipole moment components. Because *µ_a_* is predicted to be much larger than *µ_b_*, we initially looked for *R*-branch *a*-type transitions. A series of intense lines separated by approximately *B* + *C* and following the expected pattern for ^a^R transitions was observed in the broadband rotational spectrum. They all showed nuclear quadrupole coupling splittings arising from the ^14^N nucleus. Each rotational transition was also split into A and E components (see Figure 4) arising from the interaction between the methyl internal rotation and the overall rotation of the molecule. The hydrogen bond between the hydroxyl and nitro groups in 4MNP-1 impedes the rotation of the –OH group, hence there is no splitting due to this motion. From initial fits of the A torsional components of *a*-type transitions using Pickett’s *spfit* program [54] and Watson’s S-reduced Hamiltonian [55], preliminary rotational constants were determined and further *a*-type and *b*-type transitions were assigned. Many of the most intense quadrupole hyperfine components were blended in the broadband rotational spectrum. Therefore, further measurements were performed, taking advantage of the higher resolution of the Fabry–Pérot Fourier transform microwave (FTMW) spectrometer (Figure 5). A total of 327 *a*- and *b*-type lines combining A and E transitions with fully resolved nuclear quadrupole coupling splittings were measured, with an estimated measurement accuracy of 4 kHz.

Internal rotation splittings can be treated only using appropriate theoretical models. We performed two fits using two different spectral fitting programs, the *XIAM* [56] and *BELGI-C_s_-hyperfine* [57] codes, which allowed us to treat the rotational spectrum of internal rotation combined with a ^14^N quadrupole hyperfine structure. *BELGI-C_s_-hyperfine* uses the rho axis method (RAM), which is discussed in detail in refs. [58,59]. The rotational constants and the NQCCs obtained from the fit in the RAM system were subsequently transformed into the principal axis system (PAM) with the rotation matrix of *θ*_RAM_ angle around the *z*-axis, using the equations described in ref. [58]. The *XIAM* code uses the combined axis method, wherein the rotor is first set up in the PAM system and then transformed into the RAM system to eliminate the Coriolis coupling terms. In the RAM system, *XIAM* calculates the eigenvalues in the product basis of the symmetric top functions for the overall rotation and the planar rotor functions for the methyl internal rotation, and then transforms the eigenvalue matrix back to the PAM system. One difference between the two methods is that the *XIAM* code fits mainly low-order terms of the Hamiltonian, while *BELGI-C_s_-hyperfine* has more higher-order terms coupling the internal and global rotation. Furthermore, *XIAM* can fit each torsional state (*v_t_* = 0) by itself only and does not include interaction terms between different torsional states. *BELGI-C_s_-hyperfine* considers a whole set of torsional states (truncated at *v*_t_ = 8) in the Hamiltonian matrix, allowing it to obtain better root-mean-square (rms) deviation, especially for the E species of internal rotors with low torsional barriers. *XIAM* is faster and is convenient for spectral assignment, but the better rms deviation and predictive power has made *BELGI-C_s_-hyperfine* a good complement to *XIAM* [19].

For 4MNP-1, using the *XIAM* code to determine a linear combination of the rotational constants *A*, *B,* and *C*, the quartic centrifugal distortion constants *D_J_* and *D_JK_*, the ^14^N NQCCs *χ_aa_* and *χ_bb_*− *χ_cc_*, the barrier to internal rotation *V*_3_, the angle ∠(*i*,*a*) between the *a* principal inertial axis and the internal rotor axis, and three higher-order terms $D_{p_{i}^{2}J}$, $D_{p_{i}^{2}K}$, and $D_{p_{i}^{2}-}$ enabled us to reproduce the experimental spectra to an rms deviation of 7.8 kHz for 327 lines (Table 2). The internal rotation constant *F*_0_ is correlated to *V*_3_ and was fixed to the calculated value. The reduced constant *F =* ħ^2^/2*rI_α_* and the rotation-torsion coupling constant *ρ* were derived parameters.

For the *BELGI-C_s_-hyperfine* fit, presented in Table 3 in the RAM system, the same dataset of 327 hyperfine components were fitted with an rms of 3.9 kHz, floating *A*, *B,* and *C*, *Δ_K_*, *Δ_JK_*, the ^14^N NQCCs *χ_aa_* and *χ_bb_* (*χ_cc_* = −*χ_aa_*−*χ_bb_*, following Laplace condition), *V*_3_, and *ρ*. The use of the non-principal axis system in the *BELGI-C_s_-hyperfine* fit requires an additional parameter to be fitted, *D_ab_*, which is directly related to the *θ*_RAM_ angle between the RAM and PAM systems. Two higher-order terms were used, *c_2_* and *Δ_ab_*, which multiply the operators (1 − cos 3α)$\left(P_{b}^{2}-P_{c}^{2}\right)$ and {$P_{a}$,$P_{b}$}$P_{a}^{2}$, respectively. The rms deviation is within the measurement accuracy of 4 kHz. For comparison with the *XIAM* results, some parameters were converted to the PAM system and are also presented in Table 2. The *XIAM* and *BELGI-C_s_-hyperfine* parameters agree very well with each other. The frequency list and residuals of both fits are available in Table S5 of the Supplementary Materials.

## 3. Discussion and Conclusions

The most stable conformer of 4MNP was assigned, and global fits consisting of 168 A species and 159 E species transitions, including hyperfine components, were performed using two computer codes, *XIAM* and *BELGI-C_s_-hyperfine*. The *XIAM* fit has a standard deviation of 7.8 kHz, which is reduced to 3.9 kHz, consistent with measurement accuracy, using *BELGI-C_s_-hyperfine*. We can see from Table 2 that the *XIAM* and *BELGI-C_s_-hyperfine* parameters are in good agreement with each other.

Ab initio and DFT calculations were carried out to guide the analysis of the rotational spectrum of 4MNP. All levels of theory predict equilibrium rotational constants close to the experimental ground state ones, probably because of the rigidity of the aromatic ring. This was confirmed by running additional calculations with a series of different methods and basis sets for benchmarking purposes, including MP2 [37], coupled cluster methods with a double excitation model (CCSD) [60], and the functionals M06-2X [61], ωB97X-D [62], MN15 [63], and PBE [64] (see Table S6). The NQCCs obtained from Bailey’s method or from B3LYP-D3BJ and B3PW91 calculations are in good agreement with the experimental ones, but those from MP2 have an average deviation of 44.6%, showing that this method is not reliable for predicting the electronic density around the ^14^N nucleus of an –NO_2_ group.

The NQCCs of 4MNP-1, and specifically *χ_cc_*, can be compared with those from related molecules. The *c* principal inertial axis is perpendicular to the aromatic ring, as is the *z* quadrupole axis of the ^14^N atom. Therefore, *χ_cc_* coincides with *χ*_zz_ and provides information on the electric field gradient along an axis perpendicular to the molecular plane. Our value of *χ_cc_* = 0.885(17) MHz is effectively the same as those determined for nitrobenzene (*χ_cc_* = 0.8394(36) MHz) [65] and ortho-nitrophenol (*χ_cc_* = 0.886(3) MHz) [65]. The introduction of a hydroxyl group going from nitrobenzene to ortho-nitrophenol causes a very small change in the ^14^N electronic environment. No changes are observed going from ortho-nitrophenol to 4MNP-1, showing that the methyl group has no influence on the electronic environment of the ^14^N of the nitro group. Considering the definition of *χ*_zz_ and its relation to the unbalanced electrons in a *p_z_* orbital, its positive value indicates that there is an excess of electron density along the *z* axis of the ^14^N nucleus [66,67]. 

The inertial defect of 4MNP-1 is calculated to be −3.53815(48) uÅ^2^ from the *XIAM* fit. This value is consistent with a planar molecule in which only the hydrogen atoms of the methyl group are out of plane. The planar moment perpendicular to the *ab* plane, *P_cc_*, has a value of 1.76908(24) uÅ^2^. This is similar to the expected value of 1.625 uÅ^2^ for two hydrogens out of plane with C–H distances of 1.1 Å and ∠HCH of 109.28° [66] and is comparable to the *P_cc_* of other molecules wherein the only contribution to *P_cc_* arises from out-of-plane methyl hydrogens [68].

The *V*_3_ torsional barriers obtained by *XIAM* and *BELGI-C_s_-hyperfine* are 106.4456(8) cm^−1^ and 105.995(14) cm^−1^, respectively. The agreement between the values of *V*_3_ from *XIAM* and *BELGI-Cs-hyperfine* is extremely good, given the differences existing between the two methods. The barrier height for methyl internal rotation depends on steric interactions and electronic effects [17]. The methyl group in 4MNP is sufficiently far away from the –OH and –NO_2_ groups that steric interactions are unlikely, and this is reflected in the low barrier height. However, although the barrier is relatively low, it is higher than those of 18.39(3) cm^−1^ for the related 4-methylphenol (*p*-cresol) [69] and 6.7659(24) cm^−1^ for *meta*-nitrotoluene [44], which have their methyl groups in the same relative position, with respect to the –OH and –NO_2_ groups, as 4MNP-1. Other six-membered aromatic molecules where the methyl group is in para or meta with respect to a single substituent also have lower barriers than 4MNP-1 [17]. In 4MNP-1, the –NO_2_ group has negative inductive and mesomeric effects, removing electron density from the aromatic ring, while the –OH group has a positive mesomeric effect and donates electron density to the ring. Clearly the methyl group is “sensing” the presence of two other substituents in the ring, and changes in the electronic distribution induced by them (and the hydrogen bond connecting them) are very likely the cause of the higher barrier to methyl internal rotation. Further studies of toluene derivatives with two ring substituents will help get more insight into the chemical features that control the height of the barrier.

The results of this study can be used to compare the impact of the electronic environment on the methyl internal rotation barrier with other isomers of methylnitrophenol, such as 5-methyl-2-nitrophenol. The structural determination of 4MNP lays a basis to undertake future studies of the chemical reactions and atmospheric aggregation processes of 4MNP; for example, with multiple water molecules.

## 4. Materials and Methods

The rotational spectrum of 4MNP was recorded using broadband and Fabry–Pérot FTMW spectrometers. We employed first a chirped pulse FTMW spectrometer in the 2–8 GHz frequency range at King’s College London, UK [70,71]. 4MNP was purchased from Sigma-Aldrich (99% purity) and used without further purification. As 4MNP is a solid at room temperature (m.p. 305–308 K), it was heated to 369 K using a bespoke heating receptacle attached to the solenoid valve and then supersonically expanded into the vacuum chamber using neon as a carrier gas at a backing pressure of 5 bar. The vaporized molecules were excited by microwave chirped pulses of 4 µs, amplified by a travelling wave tube amplifier of 200 W, and broadcasted into the vacuum chamber using a broadband horn antenna. Once the microwave radiation stopped, the emission signal was collected in the form of a free induction decay (FID) in the time domain and converted to the frequency domain via a fast Fourier transform. The final rotational spectrum collected had 1.2 MFIDs and is shown in Figure 4.

A Fabry–Pérot FTMW spectrometer covering the 2–26.5 GHz frequency range [72] was used to determine the frequencies at higher resolutions and to expand the data set to measure transitions with higher *J* and *K* values. Solid 4MNP was put on a small piece of pipe cleaner placed in a metal tube upstream of the nozzle. The tube was also heated to about 369 K. Helium was used as the carrier gas at a backing pressure of 2 bar, flown over the 4MNP sample, and then the 4MNP–helium mixture was expanded into the cavity. A typical spectrum of the 3_1,3_ ← 2_1,2_ transition is illustrated in Figure 5.

## Figures and Tables

**Table 1:** Theoretical spectroscopic parameters of the two conformers of 4MNP calculated using the B3LYP-D3BJ, B3PW91, and MP2 methods with the 6-311++G(d,p) basis set.

Parameter		4MNP-1			4MNP-2	
	B3LYP-D3BJ	B3PW91	MP2	B3LYP-D3BJ	B3PW91	MP2
*A*^a^ (MHz)	1840.5	1855.7	1820.1	1804.8	1808.7	1816.2
*B* (MHz)	933.8	935.6	930.5	925.0	929.1	902.3
*C* (MHz)	621.9	624.4	618.1	629.4	631.5	641.8
*Δ* ^b^ *(uÅ^2^)*	−3.16	−3.12	−3.16	−23.42	−23.08	−51.03
*μ_a_*^c^ (D)	4.3	4.3	3.8	5.5	5.4	4.9
*μ_b_* (D)	−0.7	−0.8	−0.3	−3.2	−3.2	−2.7
*μ_c_* (D)	0.0	0.0	0.0	−0.2	−0.2	−0.2
*χ_aa_*^d^ (MHz)	−0.76	−0.72	−0.34	−0.76	−0.73	−0.39
*χ_bb_* (MHz)	0.02	0.01	−0.16	0.12	0.10	−0.01
*χ_cc_* (MHz)	0.74	0.71	0.50	0.65	0.62	0.38
*V*_3_^e^ (cm^−1^)	119.6	114.7	89.4	31.3	28.8	28.0
Δ*E* ^f^ (kJ mol^−1^)	0.0	0.0	0.0	44.0	45.5	27.1
Δ*E*_0_^g^ (kJ mol^−1^)	0.0	0.0	0.0	43.1	44.3	31.2

^a^ *A*, *B,* and *C* are the rotational constants; ^b^ Inertial defect calculated using *Δ* = *I_c_* − *I_a_* − *I_b_*; ^c^ *μ_a_*, *μ_b_*, and *μ_c_* are the dipole moment components; ^d^ *χ_aa_*, *χ_bb_*, and *χ_cc_* are the nuclear quadrupole coupling constants; ^e^ *V*_3_ is the barrier to methyl internal rotation; ^f^ Δ*E* is the relative energy; ^g^ Δ*E*_0_ is the relative energy including the zero-point corrections.

**Table 2:** Molecular parameters of 4MNP-1 in the PAM system obtained from fits with *XIAM* and *BELGI-C_s_-hyperfine*.

Parameter	Unit	*XIAM*	*BELGI* ^a^	Calc.
*A*	MHz	1841.7616 (26) ^b^	1841.8415961 (43)	1837.267 ^c^
*B*	MHz	932.06085 (19)	932.3075 (47)	925.714 ^c^
*C*	MHz	621.56246 (20)	621.53031 (89)	618.216 ^c^
*D_J_*	kHz	0.0076 (11)	-	0.05921 ^c^
*D_JK_*	kHz	0.0153 (58)	-	−0.06727 ^c^
*F_0_*	GHz	160.336 ^d^	160.34966 (44)	160.336 ^e^
*F*	GHz	161.9023 ^f^	161.9023 ^g^	-
*V*_3_	cm^−1^	106.4456 (8)	105.995 (14)	117.8
ρ	unitless	0.0100 ^f^	0.0099412 (28)	-
$D_{p_{i}^{2}J}$	kHz	−20.331 (88)	-	-
$D_{p_{i}^{2}K}$	MHz	0.1158 (36)	-	-
$D_{p_{i}^{2}-}$	kHz	−17.9 (2)	-	-
*χ_aa_*	MHz	−0.8150 (43)	−0.811 (27)	−0.7053 ^h^
*χ_bb_*	MHz	−0.0702 (70) ^i^	−0.075 (50)	0.0379 ^h^
*χ_cc_*	MHz	0.885 (17) ^i^	0.89 (12)	0.6674 ^h^
∠(*i*,*a*)	°	34.4141 (12)	34.37469 (49)	34.40
∠(*i*,*b*)	°	55.5859 (12)	55.62532 (49)	55.61
∠(*i*,*c*)	°	90.0 ^j^	90.0 ^j^	89.00
*rms* ^k^	kHz	7.8	3.9	-
*N_A_/N_E_/N_hf_* ^l^		168/159/327	168/159/327	-

^a^ Obtained by the RAM to PAM transformation. ^b^ Statistical uncertainties are given as one standard uncertainty in units of the last digit. ^c^ Ground state rotational constants and centrifugal distortion constants from anharmonic frequency calculations at the B3LYP-D3BJ/6-311++G(d,p) level of theory. ^d^ Fixed to the calculated value. ^e^ From geometry optimization. ^f^ Derived parameter. ^g^ Fixed to the value of the *XIAM* fit. ^h^ Calculated at the B3PW91/6-311+G(d,p)//MP2/6-311++G(d,p) levels of theory, see text. ^i^ Derived from *χ_bb_* − *χ_cc_* = −0.9554(97) MHz. ^j^ Fixed due to symmetry. ^k^ Root-mean-square deviation of the fit. ^l^ Number of A and E species transitions as well as number of the nuclear quadrupole hyperfine components.

**Table 3:** Spectroscopic constants of 4MNP-1 in the RAM system obtained using the program *BELGI-C_s_-hyperfine*.

Parameter	Unit	Value	Operator ^a^
*A*	MHz	1744.2236 (35)	$P_{a}^{2}$
*B*	MHz	1029.6800 (40)	$P_{b}^{2}$
*C*	MHz	621.53031 (89)	$P_{c}^{2}$
*D_ab_*	MHz	281.1730 (48)	$\{P_{a}$$,P_{b}$}
*Δ* *_J_*	kHz	0.0	$-P^{4}$
*Δ* *_K_*	kHz	−0.576 (33)	$-P_{a}^{4}$
*Δ* *_JK_*	kHz	0.211 (11)	$-P^{2}P_{a}^{2}$
*δ_J_*	kHz	0.0	$-2P^{2}(P_{a}^{2}-P_{c}^{2})$
*δ_K_*	kHz	0.0	$-\left\{P_{a}^{2},(P_{a}^{2}-P_{c}^{2})\right\}$
*χ_aa_*	MHz	−0.630 (46)	-
*χ_bb_*	MHz	−1.140 (46)	-
*χ_ab_*	MHz	−1.515 (57)	-
*V_3_*	cm^−1^	105.995 (14)	(1/2)(1 − cos 3α)
*ρ*	unitless	0.0099412 (28)	$P_{a}p_{\alpha }$
*F*	GHz	161.9023 ^b^	${\left(p_{\alpha }-\rho P_{a}\right)}^{2}$
*c_2_*	MHz	−0.0842 (25)	$\left(1-\cos\;3\alpha )(P_{b}^{2}-P_{c}^{2}\right)$
*Δ* *_ab_*	MHz	0.15048 (56)	$\{P_{a}$ $,P_{b}$ $\}P_{a}^{2}$
*rms* ^c^	kHz	3.9	-
*N_A_/N_E_/N_hf_* ^d^		168/159/327	-

^a^ All parameters refer to the rho axis system and cannot be directly compared to those referring to the principal axis system. $P_{a}$, $P_{b}$, and $P_{c}$ are the components of the overall rotation angular momentum; $p_{\alpha }$ is the angular momentum conjugate to the internal rotation angle α. {u,v} is the anti-commutator uv + vu. The product of the parameter and operator from a given row yields the term actually used in the vibration-rotation-torsion Hamiltonian, except for F, ρ, and *A,* which occur in the Hamiltonian in the form $\;F{\left(p_{\alpha }-\rho P_{a}\right)}^{2}+AP_{a}^{2}$, where *F = ħ^2^/2rI_α_*. Statistical uncertainties are shown as one standard uncertainty in units of the last digit. The NQCCs are defined in *BELGI-Cs-hyperfine* by a factor of two greater compared with their definitions in the *XIAM* program. ^b^ Fixed to the value obtained from *XIAM* fit. ^c^ Root-mean-square deviation of the fit. ^d^ Number of A and E species transitions as well as the nuclear quadrupole hyperfine components.

## Data Availability

Data is contained within the article and Supplementary Materials.

## Supplementary Materials

The following supporting information can be downloaded at: https://www.mdpi.com/article/10.3390/molecules28052153/s1, Figure S1: Potential energy curve of 4MNP-1 by varying the dihedral angle *α* = ∠(C3C4C9H14), corresponding to a rotation of the methyl group about the C4–C9 bond; Table S1: Nuclear coordinates of conformer 4MNP-1; Tables S2–S4: Fourier coefficients; Table S5: Frequency list and residuals obtained with the *XIAM* and *BELGI*-*C_s_*-*hyperfine* programs; Table S6: Rotational constants of 4MNP-1 calculated at different levels of theory.
