# Supplementary material for: Revealing Internal Rotation and 14N Nuclear Quadrupole Coupling in the Atmospheric Pollutant 4-Methyl-2-nitrophenol: Interplay of Microwave Spectroscopy and Quantum Chemical Calculations
Source: Molecules. 2023 Feb 24;28(5):2153. doi: 10.3390/molecules28052153 (PMC10004196; doi:10.3390/molecules28052153)
Supplement: Supplementary file 1 [file molecules-28-02153-s001.zip › molecules-2223533-supplementary.pdf]

# Supplementary Materials

## Revealing internal rotation and $^{14}N$ nuclear quadrupole coupling in the atmospheric pollutant 4-methyl-2-nitrophenol: interplay of microwave spectroscopy and quantum chemical calculations

Shefali Baweja[1,†], Eleonore Antonelli[2,†], Safia Hussain[1], Antonio Fernández-Ramos[3], Isabelle Kleiner[4], Ha Vinh Lam Nguyen[2,5,*], M. Eugenia Sanz[1,*]

[1] Department of Chemistry, King's College London, Britannia House, 7 Trinity Street, London SE1 1DB, UK

[2] Univ Paris Est Créteil and Université Paris Cité, CNRS, LISA, F-94010 Créteil, France

[3] Departamento de Química Física and Centro Singular de Investigación en Química Biolóxica e Materiais Moleculares (CIQUS), Jenaro de la Fuente s/n, Universidad de Santiago de Compostela, 15782 Santiago de Compostela, Spain

[4] Université Paris Cité and Univ Paris Est Créteil, CNRS, LISA, F-75013 Paris, France

[5] Institut Universitaire de France (IUF), 1 rue Descartes, F-75231 Paris Cedex 05, France

[†] These authors contributed equally.

**Table S1.** Nuclear coordinates in the inertial principal axis system for conformer 4MNP-1 calculated using the MP2, B3LYP-D3BJ and B3PW91 methods with the 6-311++G(d,p) basis set. The atom numbers correspond to Figure 1.

| | MP2 | | | B3LYP-D3BJ | | | B3PW91 | | |
|---|---|---|---|---|---|---|---|---|---|
| | ***a*** **/ Å** | ***b*** **/ Å** | ***c*** **/ Å** | ***a*** **/ Å** | ***b*** **/ Å** | ***c*** **/ Å** | ***a*** **/ Å** | ***b*** **/ Å** | ***c*** **/ Å** |
| **C1** | 0.300712 | 1.231849 | –0.026556 | 0.298163 | 1.230874 | 0.000002 | 0.302240 | 1.229639 | 0.000002 |
| **C2** | 0.381686 | –0.174675 | 0.038264 | 0.382630 | –0.177752 | 0.000050 | 0.382488 | –0.178122 | 0.000006 |
| **C3** | –0.766623 | –0.981366 | 0.016070 | –0.770294 | –0.977537 | 0.000086 | –0.769920 | –0.974955 | 0.000006 |
| **C4** | –2.035602 | –0.407149 | 0.019758 | –2.029266 | –0.408915 | 0.000028 | –2.028288 | –0.406630 | 0.000004 |
| **C5** | –2.124004 | 0.998978 | –0.032945 | –2.111135 | 0.998758 | –0.000088 | –2.105697 | 0.999838 | –0.000002 |
| **C6** | –0.986917 | 1.797329 | –0.027739 | –0.985977 | 1.797353 | –0.000126 | –0.980463 | 1.796080 | –0.000005 |
| **O7** | 1.349566 | 2.077662 | –0.035666 | 1.348043 | 2.058792 | 0.000105 | 1.351759 | 2.049029 | 0.000001 |
| **N8** | 1.680139 | –0.844664 | 0.001435 | 1.670324 | –0.842283 | –0.000015 | 1.665805 | –0.842371 | 0.000000 |
| **C9** | –3.279737 | –1.258803 | 0.046078 | –3.278570 | –1.251263 | 0.000058 | –3.275339 | –1.245967 | 0.000000 |
| **O10** | 2.697743 | –0.140663 | 0.140485 | 2.695836 | –0.130642 | 0.000011 | 2.685683 | –0.132570 | 0.000008 |
| **O11** | 1.714719 | –2.063899 | –0.137112 | 1.714357 | –2.061423 | –0.000108 | 1.709205 | –2.055780 | –0.000012 |
| **H12** | 2.149477 | 1.521524 | 0.008605 | 2.156146 | 1.501313 | 0.000085 | 2.152525 | 1.479089 | 0.000001 |
| **H13** | –0.639153 | –2.058722 | 0.039934 | –0.639830 | –2.051025 | 0.000130 | –0.638254 | –2.050239 | 0.000010 |
| **H14** | –3.039484 | –2.310310 | –0.129992 | –3.037589 | –2.315178 | 0.000676 | –3.036506 | –2.311062 | 0.000020 |
| **H15** | –3.781521 | –1.182899 | 1.016517 | –3.893104 | –1.044612 | 0.881182 | –3.890672 | –1.040287 | 0.881620 |
| **H16** | –3.987479 | –0.934995 | –0.722808 | –3.892533 | –1.045529 | –0.881687 | –3.890643 | –1.040315 | –0.881647 |
| **H17** | –3.103135 | 1.473432 | –0.043698 | –3.088298 | 1.470359 | –0.000203 | –3.082437 | 1.475595 | –0.000002 |
| **H18** | –1.057834 | 2.880448 | –0.068070 | –1.058297 | 2.877468 | –0.000227 | –1.052648 | 2.877583 | –0.000011 |

**Table S2.** Coefficients of the one-dimensional Fourier expansion for the potential energy curves of 4MNP-1 corresponding to Figure S1 calculated at the MP2/6-311++G(d,p) and B3LYP-D3BJ/6-311++G(d,p) levels of theory. The potential is expanded as $V(\alpha) = \sum_{i=0}^{n} a_i\ f_i$.

| | | MP2 | | B3LYP-D3BJ | |
|---|---|---|---|---|---|
| **i** | $\mathbf{f_i}$ | $\mathbf{a_i}$ **/ Hartree** | $\mathbf{a_i}$ **/ cm$^{-1}$** | $\mathbf{a_i}$ **/ Hartree** | $\mathbf{a_i}$ **/ cm$^{-1}$** |
| 0 | 1 | −549.977643082 | | −551.489572611 | |
| 1 | cos(3α) | −0.000197646 | 43.4 | −0.000268241 | 58.9 |
| 2 | cos(6α) | 0.000047188 | 10.4 | 0.000009863 | 2.2 |
| 3 | sin(3α) | 0.000032343 | 7.1 | / | / |

**Figure S1.** Potential energy curve of 4MNP-1 obtained at the B3LYP-D3BJ/6-311++G(d,p) (black) and MP2/6-311++G(d,p) (blue) levels of theory by varying the dihedral angle $\alpha$ = ∠(C3,C4,C9,H14), corresponding to a rotation of the methyl group about the C4-C9 bond.

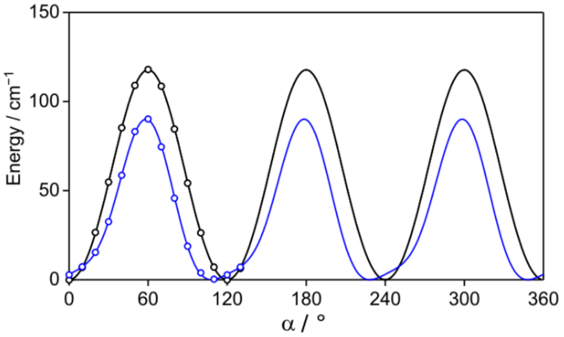


**Table S3.** First eigenvalues (in $cm^{-1}$) after diagonalization of the one-dimensional ($E_{1D}$) and two-dimensional ($E_{2D}$) potentials of Figure 2. All listed $E_{2D}$ levels correspond to excitations of the methyl group torsion.

| Level | $E_{1D}$ | $E_{2D}$ |
|---|---|---|
| 0 | 33.24 | 304.66 |
| 1 | 34.05 | 305.55 |
| 2 | 34.05 | 305.55 |
| 3 | 89.14 | 358.89 |
| 4 | 89.14 | 358.89 |
| 5 | 100.09 | 370.77 |
| 6 | 123.26 | 392.19 |
| 7 | 148.99 | 418.51 |
| 8 | 148.99 | 418.51 |
| 9 | 195.51 | 465.21 |

| Level | $E_{1D}$ | $E_{2D}$ |
|---|---|---|
| 10 | 195.51 | 465.21 |
| 11 | 253.79 | 523.63 |
| 12 | 254.08 | 523.88 |
| 13 | 323.72 | 593.62 |
| 14 | 323.72 | 593.62 |
| 15 | 404.58 | 674.54 |
| 16 | 404.58 | 674.54 |
| 17 | 496.40 | 766.42 |
| 18 | 496.40 | 766.42 |

The one-dimensional levels were obtained by the diagonalization of the $V_3$ potential whereas the two-dimensional levels involved the diagonalization of the Schrödinger equation with a potential of the type:

$$V(\phi_1,\phi_2) = a_0 + a_3(\phi_1) + \sum_{k=1}^{6} b_k \cos(k\phi_2) + \sum_{k=1}^{6} c_{3,k} \cos(3\phi_1)\cos(k\phi_2) + \sum_{k=1}^{6} d_{3,k} \sin(3\phi_1)\sin(k\phi_2) \quad \text{(S1)}$$

**Table S4.** Coefficients of the Fourier series of equation S1.

| | |
|---|---|
| $a_0$ | 3159.0 |
| $a_3$ | 19.19 |
| $b_1$ | 1776.1 |
| $b_2$ | –1197.1 |
| $b_3$ | 120.08 |
| $b_4$ | –20.38 |
| $b_5$ | –8.88 |
| $b_6$ | 1.25 |
| $c_{3,1}$ | –19.44 |
| $c_{3,2}$ | 13.67 |
| $c_{3,3}$ | –3.79 |
| $c_{3,4}$ | 1.75 |
| $c_{3,5}$ | 0.23 |
| $c_{3,6}$ | –2.06 |
| $d_{3,1}$ | 1.36 |
| $d_{3,2}$ | –8.37 |
| $d_{3,3}$ | –0.081 |
| $d_{3,4}$ | –1.32 |
| $d_{3,5}$ | –0.89 |
| $d_{3,6}$ | –1.59 |

**Table S5.** Observed frequencies ($\nu_{obs}$) of the A and E species of 4MNP-1. The $\nu_{obs} - \nu_{calc}$ residuals are obtained with the *XIAM* and *BELGI-C$_s$-hyperfine* programs.

| *J* | *K$_a$* | *K$_c$* | *F* | *J* | *K$_a$* | *K$_c$* | *F* | *Species* | $\nu_{obs}$ / MHz | $\nu_{obs} - \nu_{calc}$ *XIAM*/ kHz | $\nu_{obs} - \nu_{calc}$ *BELGI*/ kHz |
|---|---|---|---|---|---|---|---|---|---|---|---|
| Upper level | | | | Lower level | | | | | | | |
| 2 | 0 | 2 | 2 | 1 | 0 | 1 | 2 | A | 3040.9781 | 1.7 | –1 |
| 2 | 0 | 2 | 1 | 1 | 0 | 1 | 0 | A | 3041.0739 | 1.6 | –1 |
| 2 | 0 | 2 | 2 | 1 | 0 | 1 | 1 | A | 3041.2236 | 3.0 | 2 |
| 2 | 0 | 2 | 3 | 1 | 0 | 1 | 2 | A | 3041.2749 | 1.4 | 0 |
| 2 | 0 | 2 | 2 | 1 | 0 | 1 | 2 | E | 3027.1453 | –9.9 | –1 |
| 2 | 0 | 2 | 2 | 1 | 0 | 1 | 1 | E | 3027.3654 | –28.2 | 1 |
| 2 | 0 | 2 | 3 | 1 | 0 | 1 | 2 | E | 3027.4484 | 1.5 | 1 |
| 2 | 1 | 1 | 2 | 1 | 1 | 0 | 1 | A | 3418.7926 | 16.4 | 12 |
| 2 | 1 | 1 | 3 | 1 | 1 | 0 | 2 | A | 3419.0219 | 3.0 | 0 |
| 2 | 1 | 1 | 1 | 1 | 1 | 0 | 0 | A | 3419.4051 | 0.7 | –1 |
| 2 | 1 | 1 | 2 | 1 | 1 | 0 | 1 | E | 3316.6403 | 17.3 | 2 |
| 2 | 1 | 1 | 3 | 1 | 1 | 0 | 2 | E | 3316.8156 | 1.8 | 2 |
| 2 | 1 | 1 | 1 | 1 | 1 | 0 | 0 | E | 3317.0695 | –22.5 | 0 |
| 2 | 1 | 2 | 2 | 1 | 1 | 1 | 2 | A | 2796.9410 | 1.2 | 2 |
| 2 | 1 | 2 | 2 | 1 | 1 | 1 | 1 | A | 2796.9598 | –1.4 | –2 |
| 2 | 1 | 2 | 3 | 1 | 1 | 1 | 2 | A | 2797.2258 | 1.5 | 2 |
| 2 | 1 | 2 | 1 | 1 | 1 | 1 | 0 | A | 2797.3462 | –4.1 | –1 |
| 2 | 1 | 2 | 1 | 1 | 1 | 1 | 1 | A | 2797.4047 | 0.9 | 0 |
| 2 | 1 | 2 | 2 | 1 | 1 | 1 | 1 | E | 2896.5013 | –21.2 | –2 |
| 2 | 1 | 2 | 3 | 1 | 1 | 1 | 2 | E | 2896.8367 | 0.0 | –4 |
| 2 | 1 | 2 | 1 | 1 | 1 | 1 | 1 | E | 2896.8913 | –32.8 | –5 |
| 2 | 1 | 2 | 1 | 1 | 1 | 1 | 0 | E | 2897.1141 | 49.8 | 5 |
| 3 | 0 | 3 | 3 | 2 | 0 | 2 | 3 | A | 4412.6220 | 1.2 | –1 |
| 3 | 0 | 3 | 3 | 2 | 0 | 2 | 2 | A | 4412.9153 | –2.5 | –3 |
| 3 | 0 | 3 | 2 | 2 | 0 | 2 | 1 | A | 4412.9384 | 3.6 | 2 |
| 3 | 0 | 3 | 4 | 2 | 0 | 2 | 3 | A | 4412.9756 | 0.0 | –1 |
| 3 | 0 | 3 | 2 | 2 | 0 | 2 | 2 | A | 4413.3951 | –1.8 | –1 |
| 3 | 0 | 3 | 3 | 2 | 0 | 2 | 3 | E | 4404.1628 | –6.6 | –1 |
| 3 | 0 | 3 | 3 | 2 | 0 | 2 | 2 | E | 4404.4676 | 6.5 | 2 |
| 3 | 0 | 3 | 2 | 2 | 0 | 2 | 1 | E | 4404.4782 | –3.0 | 1 |
| 3 | 0 | 3 | 4 | 2 | 0 | 2 | 3 | E | 4404.5172 | –3.2 | –3 |
| 3 | 0 | 3 | 2 | 2 | 0 | 2 | 2 | E | 4404.9464 | 11.5 | 0 |
| 3 | 1 | 2 | 3 | 2 | 1 | 1 | 2 | A | 5081.0913 | 4.6 | 0 |
| 3 | 1 | 2 | 4 | 2 | 1 | 1 | 3 | A | 5081.1113 | –56.1 | –2 |
| 3 | 1 | 2 | 2 | 2 | 1 | 1 | 1 | A | 5081.2045 | 4.2 | 0 |
| 3 | 1 | 2 | 3 | 2 | 1 | 1 | 2 | E | 5018.7405 | 3.7 | 1 |
| 3 | 1 | 2 | 4 | 2 | 1 | 1 | 3 | E | 5018.8011 | 2.6 | –1 |
| 3 | 1 | 2 | 2 | 2 | 1 | 1 | 1 | E | 5018.8331 | 14.7 | 10 |
| 3 | 1 | 3 | 3 | 2 | 1 | 2 | 3 | A | 4157.3121 | –0.4 | 0 |
| 3 | 1 | 3 | 3 | 2 | 1 | 2 | 2 | A | 4157.5965 | –0.6 | 0 |
| 3 | 1 | 3 | 4 | 2 | 1 | 2 | 3 | A | 4157.6860 | 0.4 | 2 |
| 3 | 1 | 3 | 2 | 2 | 1 | 2 | 2 | A | 4158.0992 | –1.5 | 0 |

| | | | | | | | | | | | |
|---|---|---|---|---|---|---|---|---|---|---|---|
| 3 | 1 | 3 | 3 | 2 | 1 | 2 | 3 | E | 4197.9070 | 1.5 | 0 |
| 3 | 1 | 3 | 3 | 2 | 1 | 2 | 2 | E | 4198.1602 | –3.5 | 1 |
| 3 | 1 | 3 | 4 | 2 | 1 | 2 | 3 | E | 4198.2708 | 1.2 | 0 |
| 3 | 1 | 3 | 2 | 2 | 1 | 2 | 2 | E | 4198.6504 | –4.8 | 0 |
| 3 | 2 | 1 | 3 | 2 | 2 | 0 | 2 | A | 4911.1013 | 7.6 | 4 |
| 3 | 2 | 1 | 4 | 2 | 2 | 0 | 3 | A | 4911.3156 | 8.4 | 7 |
| 3 | 2 | 1 | 2 | 2 | 2 | 0 | 1 | A | 4911.4477 | 4.8 | 4 |
| 3 | 2 | 1 | 4 | 2 | 2 | 0 | 2 | E | 4792.2381 | 3.7 | –12 |
| 3 | 2 | 1 | 3 | 2 | 2 | 0 | 2 | E | 4792.2959 | 13.2 | 4 |
| 3 | 2 | 1 | 2 | 2 | 2 | 0 | 1 | E | 4792.6468 | –3.3 | 4 |
| 3 | 2 | 2 | 3 | 2 | 2 | 1 | 2 | A | 4661.9065 | 3.0 | 0 |
| 3 | 2 | 2 | 4 | 2 | 2 | 1 | 3 | A | 4662.1713 | 6.3 | 5 |
| 3 | 2 | 2 | 2 | 2 | 2 | 1 | 1 | A | 4662.3088 | –1.6 | –2 |
| 3 | 2 | 2 | 3 | 2 | 2 | 1 | 2 | E | 4791.2494 | –4.5 | –1 |
| 3 | 2 | 2 | 4 | 2 | 2 | 1 | 3 | E | 4791.5085 | 9.6 | 4 |
| 3 | 2 | 2 | 2 | 2 | 2 | 1 | 1 | E | 4791.6417 | –0.6 | –13 |
| 4 | 0 | 4 | 4 | 3 | 0 | 3 | 4 | A | 5677.0971 | 0.1 | –1 |
| 4 | 0 | 4 | 4 | 3 | 0 | 3 | 3 | A | 5677.4441 | –7.8 | –8 |
| 4 | 0 | 4 | 3 | 3 | 0 | 3 | 2 | A | 5677.4957 | 10.1 | 9 |
| 4 | 0 | 4 | 5 | 3 | 0 | 3 | 4 | A | 5677.5114 | 6.5 | 6 |
| 4 | 0 | 4 | 3 | 3 | 0 | 3 | 3 | A | 5677.9632 | –1.5 | –1 |
| 4 | 0 | 4 | 4 | 3 | 0 | 3 | 4 | E | 5672.1561 | –3.3 | –1 |
| 4 | 0 | 4 | 4 | 3 | 0 | 3 | 3 | E | 5672.5121 | 1.7 | –1 |
| 4 | 0 | 4 | 3 | 3 | 0 | 3 | 2 | E | 5672.5442 | –2.1 | –1 |
| 4 | 0 | 4 | 5 | 3 | 0 | 3 | 4 | E | 5672.5643 | –0.6 | 0 |
| 4 | 0 | 4 | 3 | 3 | 0 | 3 | 3 | E | 5673.0254 | 5.3 | 0 |
| 4 | 1 | 3 | 4 | 3 | 1 | 2 | 3 | A | 6673.8627 | 2.7 | –1 |
| 4 | 1 | 3 | 5 | 3 | 1 | 2 | 4 | A | 6673.9164 | 3.1 | –1 |
| 4 | 1 | 3 | 3 | 3 | 1 | 2 | 2 | A | 6673.9266 | 5.0 | 1 |
| 4 | 1 | 3 | 4 | 3 | 1 | 2 | 3 | E | 6624.8384 | 6.2 | 4 |
| 4 | 1 | 3 | 5 | 3 | 1 | 2 | 4 | E | 6624.8816 | 4.0 | 0 |
| 4 | 1 | 3 | 3 | 3 | 1 | 2 | 2 | E | 6624.8879 | 5.1 | 1 |
| 4 | 1 | 4 | 4 | 3 | 1 | 3 | 4 | A | 5483.4649 | 0.4 | 2 |
| 4 | 1 | 4 | 4 | 3 | 1 | 3 | 3 | A | 5483.8380 | 0.5 | 2 |
| 4 | 1 | 4 | 5 | 3 | 1 | 3 | 4 | A | 5483.8859 | –1.1 | 0 |
| 4 | 1 | 4 | 3 | 3 | 1 | 3 | 3 | A | 5484.3673 | –1.4 | 1 |
| 4 | 1 | 4 | 4 | 3 | 1 | 3 | 4 | E | 5498.2813 | –1.2 | 0 |
| 4 | 1 | 4 | 4 | 3 | 1 | 3 | 3 | E | 5498.6457 | –0.8 | 1 |
| 4 | 1 | 4 | 3 | 3 | 1 | 3 | 2 | E | 5498.6802 | –1.0 | 0 |
| 4 | 1 | 4 | 5 | 3 | 1 | 3 | 4 | E | 5498.6997 | –1.3 | 0 |
| 4 | 1 | 4 | 3 | 3 | 1 | 3 | 3 | E | 5499.1721 | –0.6 | 1 |
| 4 | 2 | 2 | 4 | 3 | 2 | 1 | 3 | A | 6701.1152 | –5.4 | –9 |
| 4 | 2 | 2 | 5 | 3 | 2 | 1 | 4 | A | 6701.2006 | 6.7 | 4 |
| 4 | 2 | 2 | 3 | 3 | 2 | 1 | 2 | A | 6701.2263 | 5.7 | 3 |
| 4 | 2 | 2 | 4 | 3 | 2 | 1 | 3 | E | 6527.4763 | –2.8 | –9 |
| 4 | 2 | 2 | 5 | 3 | 2 | 1 | 4 | E | 6527.5547 | 6.0 | 5 |
| 4 | 2 | 2 | 3 | 3 | 2 | 1 | 2 | E | 6527.5863 | 15.2 | 16 |
| 4 | 2 | 3 | 4 | 3 | 2 | 2 | 3 | A | 6163.4830 | 0.8 | –1 |

| | | | | | | | | | | | |
|---|---|---|---|---|---|---|---|---|---|---|---|
| 4 | 2 | 3 | 5 | 3 | 2 | 2 | 4 | A | 6163.6044 | 2.3 | 1 |
| 4 | 2 | 3 | 3 | 3 | 2 | 2 | 2 | A | 6163.6332 | 0.2 | 0 |
| 4 | 2 | 3 | 5 | 3 | 2 | 2 | 4 | E | 6346.8323 | 2.7 | –2 |
| 4 | 3 | 1 | 4 | 3 | 3 | 0 | 3 | A | 6380.0359 | 1.9 | –1 |
| 4 | 3 | 1 | 5 | 3 | 3 | 0 | 4 | A | 6380.2628 | 11.3 | 10 |
| 4 | 3 | 1 | 3 | 3 | 3 | 0 | 2 | A | 6380.3368 | –3.4 | –4 |
| 5 | 0 | 5 | 5 | 4 | 0 | 4 | 5 | A | 6893.7557 | –0.2 | –1 |
| 5 | 0 | 5 | 5 | 4 | 0 | 4 | 4 | A | 6894.1645 | 0.7 | 1 |
| 5 | 0 | 5 | 4 | 4 | 0 | 4 | 3 | A | 6894.1894 | –2.4 | –2 |
| 5 | 0 | 5 | 6 | 4 | 0 | 4 | 5 | A | 6894.2049 | –0.2 | 0 |
| 5 | 0 | 5 | 4 | 4 | 0 | 4 | 4 | A | 6894.7019 | –2.7 | –2 |
| 5 | 0 | 5 | 5 | 4 | 0 | 4 | 5 | E | 6890.1574 | –2.6 | –1 |
| 5 | 0 | 5 | 5 | 4 | 0 | 4 | 4 | E | 6890.5655 | 0.0 | –1 |
| 5 | 0 | 5 | 4 | 4 | 0 | 4 | 3 | E | 6890.5898 | –4.8 | –4 |
| 5 | 0 | 5 | 6 | 4 | 0 | 4 | 5 | E | 6890.6064 | –1.3 | –1 |
| 5 | 0 | 5 | 4 | 4 | 0 | 4 | 4 | E | 6891.1060 | 1.6 | –1 |
| 5 | 1 | 4 | 5 | 4 | 1 | 3 | 4 | A | 8158.9412 | 2.5 | –1 |
| 5 | 1 | 4 | 6 | 4 | 1 | 3 | 5 | A | 8158.9911 | 1.6 | –2 |
| 5 | 1 | 4 | 4 | 4 | 1 | 3 | 3 | A | 8158.9959 | 2.0 | –2 |
| 5 | 1 | 4 | 5 | 4 | 1 | 3 | 4 | E | 8123.4494 | 2.0 | 0 |
| 5 | 1 | 4 | 6 | 4 | 1 | 3 | 5 | E | 8123.4961 | 1.9 | –1 |
| 5 | 1 | 4 | 4 | 4 | 1 | 3 | 3 | E | 8123.4961 | –1.4 | –4 |
| 5 | 1 | 5 | 5 | 4 | 1 | 4 | 5 | A | 6778.5709 | –4.2 | –3 |
| 5 | 1 | 5 | 6 | 4 | 1 | 4 | 5 | A | 6779.0149 | –17.0 | –15 |
| 5 | 1 | 5 | 4 | 4 | 1 | 4 | 4 | A | 6779.5413 | –6.2 | –4 |
| 5 | 1 | 5 | 5 | 4 | 1 | 4 | 4 | E | 6784.1612 | –1.6 | 0 |
| 5 | 1 | 5 | 6 | 4 | 1 | 4 | 5 | E | 6784.1973 | –1.6 | 0 |
| 5 | 1 | 5 | 4 | 4 | 1 | 4 | 4 | E | 6784.7101 | 0.0 | 1 |
| 5 | 2 | 3 | 5 | 4 | 2 | 2 | 4 | A | 8489.9893 | 2.9 | 0 |
| 5 | 2 | 3 | 6 | 4 | 2 | 2 | 5 | A | 8490.0247 | 1.8 | –1 |
| 5 | 2 | 3 | 4 | 4 | 2 | 2 | 3 | A | 8490.0346 | 3.7 | 1 |
| 5 | 2 | 3 | 5 | 4 | 2 | 2 | 4 | E | 8346.9344 | 10.6 | 6 |
| 5 | 2 | 3 | 6 | 4 | 2 | 2 | 5 | E | 8346.9490 | 1.8 | –1 |
| 5 | 2 | 3 | 4 | 4 | 2 | 2 | 3 | E | 8346.9490 | –1.8 | –4 |
| 5 | 2 | 4 | 5 | 4 | 2 | 3 | 4 | A | 7622.2239 | –0.3 | 0 |
| 5 | 2 | 4 | 6 | 4 | 2 | 3 | 5 | A | 7622.2969 | 0.7 | 1 |
| 5 | 2 | 4 | 4 | 4 | 2 | 3 | 3 | A | 7622.3108 | 6.3 | 6 |
| 5 | 2 | 4 | 5 | 4 | 2 | 3 | 4 | E | 7755.6592 | –2.1 | –1 |
| 5 | 2 | 4 | 6 | 4 | 2 | 3 | 5 | E | 7755.7498 | 1.4 | –1 |
| 5 | 2 | 4 | 4 | 4 | 2 | 3 | 3 | E | 7755.7674 | 6.1 | 3 |
| 5 | 3 | 2 | 5 | 4 | 3 | 1 | 4 | A | 8094.5379 | 3.0 | 1 |
| 5 | 3 | 2 | 6 | 4 | 3 | 1 | 5 | A | 8094.6333 | 1.2 | 1 |
| 5 | 3 | 2 | 4 | 4 | 3 | 1 | 3 | A | 8094.6527 | –6.4 | –7 |
| 5 | 3 | 2 | 5 | 4 | 3 | 1 | 4 | E | 7993.8461 | 0.3 | –2 |
| 5 | 3 | 2 | 6 | 4 | 3 | 1 | 5 | E | 7993.9530 | 0.2 | 2 |
| 5 | 3 | 2 | 4 | 4 | 3 | 1 | 3 | E | 7993.9714 | –9.9 | –8 |
| 5 | 3 | 3 | 5 | 4 | 3 | 2 | 4 | A | 7920.0503 | 3.2 | 2 |
| 5 | 3 | 3 | 6 | 4 | 3 | 2 | 5 | A | 7920.1670 | 1.3 | 0 |

| | | | | | | | | | | | |
|---|---|---|---|---|---|---|---|---|---|---|---|
| 5 | 3 | 3 | 4 | 4 | 3 | 2 | 3 | A | 7920.1956 | –0.7 | –1 |
| 5 | 3 | 3 | 5 | 4 | 3 | 2 | 4 | E | 8028.4663 | 6.7 | 5 |
| 5 | 3 | 3 | 6 | 4 | 3 | 2 | 5 | E | 8028.5750 | 7.1 | 2 |
| 5 | 3 | 3 | 4 | 4 | 3 | 2 | 3 | E | 8028.6038 | 6.9 | 1 |
| 5 | 4 | 1 | 5 | 4 | 4 | 0 | 4 | A | 7926.1737 | 4.4 | 1 |
| 5 | 4 | 1 | 6 | 4 | 4 | 0 | 5 | A | 7926.3785 | 5.3 | 4 |
| 5 | 4 | 1 | 4 | 4 | 4 | 0 | 3 | A | 7926.4507 | 14.7 | 14 |
| 5 | 4 | 1 | 5 | 4 | 4 | 0 | 4 | E | 7912.3163 | 0.5 | –2 |
| 5 | 4 | 1 | 6 | 4 | 4 | 0 | 5 | E | 7912.5143 | –6.7 | –5 |
| 5 | 4 | 1 | 4 | 4 | 4 | 0 | 3 | E | 7912.5784 | –5.6 | –2 |
| 5 | 4 | 2 | 5 | 4 | 4 | 1 | 4 | A | 7917.9245 | 6.7 | 4 |
| 5 | 4 | 2 | 6 | 4 | 4 | 1 | 5 | A | 7918.1280 | 4.8 | 3 |
| 5 | 4 | 2 | 4 | 4 | 4 | 1 | 3 | A | 7918.1985 | 12.2 | 11 |
| 5 | 4 | 2 | 5 | 4 | 4 | 1 | 4 | E | 7929.4577 | 5.5 | 2 |
| 6 | 0 | 6 | 6 | 5 | 0 | 5 | 5 | A | 8109.4255 | –1.3 | 0 |
| 6 | 0 | 6 | 5 | 5 | 0 | 5 | 4 | A | 8109.4438 | –2.8 | –2 |
| 6 | 0 | 6 | 7 | 5 | 0 | 5 | 6 | A | 8109.4584 | 1.3 | 2 |
| 6 | 0 | 6 | 6 | 5 | 0 | 5 | 5 | E | 8106.6738 | –0.6 | 0 |
| 6 | 0 | 6 | 5 | 5 | 0 | 5 | 4 | E | 8106.6933 | –1.5 | –1 |
| 6 | 0 | 6 | 7 | 5 | 0 | 5 | 6 | E | 8106.7061 | 0.9 | 2 |
| 6 | 1 | 5 | 6 | 5 | 1 | 4 | 5 | A | 9509.9805 | 2.8 | 0 |
| 6 | 1 | 5 | 7 | 5 | 1 | 4 | 6 | A | 9510.0340 | 3.5 | 0 |
| 6 | 1 | 5 | 5 | 5 | 1 | 4 | 4 | A | 9510.0340 | 0.2 | –3 |
| 6 | 1 | 5 | 6 | 5 | 1 | 4 | 5 | E | 9489.4463 | 1.5 | –1 |
| 6 | 1 | 5 | 7 | 5 | 1 | 4 | 6 | E | 9489.4993 | 3.1 | 1 |
| 6 | 1 | 5 | 5 | 5 | 1 | 4 | 4 | E | 9489.4993 | 0.1 | –1 |
| 6 | 1 | 6 | 6 | 5 | 1 | 5 | 5 | A | 8051.0889 | –2.0 | 0 |
| 6 | 1 | 6 | 5 | 5 | 1 | 5 | 4 | A | 8051.1030 | –2.5 | –1 |
| 6 | 1 | 6 | 7 | 5 | 1 | 5 | 6 | A | 8051.1168 | –0.2 | 2 |
| 6 | 1 | 6 | 6 | 5 | 1 | 5 | 5 | E | 8052.3795 | –1.3 | 1 |
| 6 | 1 | 6 | 5 | 5 | 1 | 5 | 4 | E | 8052.3932 | –3.2 | –1 |
| 6 | 1 | 6 | 7 | 5 | 1 | 5 | 6 | E | 8052.4072 | –0.5 | 1 |
| 6 | 2 | 4 | 6 | 5 | 2 | 3 | 5 | A | 10211.7823 | 0.6 | –2 |
| 6 | 2 | 4 | 7 | 5 | 2 | 3 | 6 | A | 10211.8121 | 1.5 | –1 |
| 6 | 2 | 4 | 5 | 5 | 2 | 3 | 4 | A | 10211.8121 | –2.5 | –5 |
| 6 | 2 | 4 | 6 | 5 | 2 | 3 | 5 | E | 10109.9390 | 3.1 | 1 |
| 6 | 2 | 4 | 7 | 5 | 2 | 3 | 6 | E | 10109.9600 | 3.3 | 1 |
| 6 | 2 | 4 | 5 | 5 | 2 | 3 | 4 | E | 10109.9600 | 1.4 | –1 |
| 6 | 2 | 5 | 6 | 5 | 2 | 4 | 5 | A | 9032.7385 | 0.0 | 2 |
| 6 | 2 | 5 | 7 | 5 | 2 | 4 | 6 | A | 9032.7911 | 1.1 | 3 |
| 6 | 2 | 5 | 5 | 5 | 2 | 4 | 4 | A | 9032.7911 | –1.0 | 1 |
| 6 | 2 | 5 | 6 | 5 | 2 | 4 | 5 | E | 9096.7785 | –1.2 | 1 |
| 6 | 2 | 5 | 7 | 5 | 2 | 4 | 6 | E | 9096.8404 | 0.7 | 2 |
| 6 | 2 | 5 | 5 | 5 | 2 | 4 | 4 | E | 9096.8404 | –3.3 | –3 |
| 6 | 3 | 3 | 6 | 5 | 3 | 2 | 5 | A | 9899.7747 | 0.6 | 2 |
| 6 | 3 | 3 | 7 | 5 | 3 | 2 | 6 | A | 9899.8145 | –1.8 | 0 |
| 6 | 3 | 3 | 5 | 5 | 3 | 2 | 4 | A | 9899.8244 | –0.3 | 1 |
| 6 | 3 | 3 | 6 | 5 | 3 | 2 | 5 | E | 9712.9566 | 0.1 | 0 |

| | | | | | | | | | | | |
|---|---|---|---|---|---|---|---|---|---|---|---|
| 6 | 3 | 3 | 7 | 5 | 3 | 2 | 6 | E | 9713.0015 | –4.3 | –1 |
| 6 | 3 | 3 | 5 | 5 | 3 | 2 | 4 | E | 9713.0108 | –4.0 | –1 |
| 6 | 3 | 4 | 6 | 5 | 3 | 3 | 5 | A | 9494.2856 | 2.1 | 2 |
| 6 | 3 | 4 | 7 | 5 | 3 | 3 | 6 | A | 9494.3579 | 2.0 | 3 |
| 6 | 3 | 4 | 5 | 5 | 3 | 3 | 4 | A | 9494.3743 | 6.1 | 7 |
| 6 | 3 | 4 | 6 | 5 | 3 | 3 | 5 | E | 9694.9451 | 3.9 | 3 |
| 6 | 3 | 4 | 7 | 5 | 3 | 3 | 6 | E | 9695.0117 | 4.9 | 2 |
| 6 | 3 | 4 | 5 | 5 | 3 | 3 | 4 | E | 9695.0270 | 8.4 | 5 |
| 6 | 4 | 2 | 6 | 5 | 4 | 1 | 5 | A | 9576.2218 | 1.4 | 1 |
| 6 | 4 | 2 | 7 | 5 | 4 | 1 | 6 | A | 9576.3330 | –0.2 | 0 |
| 6 | 4 | 2 | 5 | 5 | 4 | 1 | 4 | A | 9576.3587 | –0.4 | 0 |
| 6 | 4 | 2 | 6 | 5 | 4 | 1 | 5 | E | 9542.7489 | –4.4 | –3 |
| 6 | 4 | 2 | 7 | 5 | 4 | 1 | 6 | E | 9542.8636 | –5.5 | –2 |
| 6 | 4 | 2 | 5 | 5 | 4 | 1 | 4 | E | 9542.8860 | –9.3 | –5 |
| 6 | 4 | 3 | 6 | 5 | 4 | 2 | 5 | A | 9540.4916 | 1.6 | 1 |
| 6 | 4 | 3 | 7 | 5 | 4 | 2 | 6 | A | 9540.6046 | –2.8 | –3 |
| 6 | 4 | 3 | 5 | 5 | 4 | 2 | 4 | A | 9540.6309 | –3.1 | –3 |
| 6 | 4 | 3 | 6 | 5 | 4 | 2 | 5 | E | 9573.1121 | 10.3 | 7 |
| 6 | 4 | 3 | 7 | 5 | 4 | 2 | 6 | E | 9573.2125 | –3.7 | –9 |
| 6 | 4 | 3 | 5 | 5 | 4 | 2 | 4 | E | 9573.2356 | –6.7 | –13 |
| 6 | 5 | 1 | 6 | 5 | 5 | 0 | 5 | A | 9495.3807 | 0.9 | –2 |
| 6 | 5 | 1 | 7 | 5 | 5 | 0 | 6 | A | 9495.5674 | 1.2 | 0 |
| 6 | 5 | 1 | 5 | 5 | 5 | 0 | 4 | A | 9495.6094 | –3.2 | –4 |
| 6 | 5 | 2 | 6 | 5 | 5 | 1 | 5 | E | 9499.6290 | 9.7 | 4 |
| 6 | 5 | 2 | 7 | 5 | 5 | 1 | 6 | E | 9499.8208 | 15.4 | 7 |
| 6 | 5 | 2 | 5 | 5 | 5 | 1 | 4 | E | 9499.8623 | 10.5 | 2 |
| 7 | 0 | 7 | 7 | 6 | 0 | 6 | 6 | A | 9335.4858 | –2.3 | –2 |
| 7 | 0 | 7 | 6 | 6 | 0 | 6 | 5 | A | 9335.5012 | –1.0 | 0 |
| 7 | 0 | 7 | 8 | 6 | 0 | 6 | 7 | A | 9335.5114 | 0.7 | 1 |
| 7 | 0 | 7 | 7 | 6 | 0 | 6 | 6 | E | 9333.2957 | –2.1 | –2 |
| 7 | 0 | 7 | 6 | 6 | 0 | 6 | 5 | E | 9333.3113 | –0.9 | 0 |
| 7 | 0 | 7 | 8 | 6 | 0 | 6 | 7 | E | 9333.3217 | 1.0 | 2 |
| 7 | 1 | 6 | 7 | 6 | 1 | 5 | 6 | A | 10747.1221 | 0.0 | –1 |
| 7 | 1 | 6 | 8 | 6 | 1 | 5 | 7 | A | 10747.1731 | 0.4 | –1 |
| 7 | 1 | 6 | 6 | 6 | 1 | 5 | 5 | A | 10747.1731 | –1.6 | –3 |
| 7 | 1 | 6 | 7 | 6 | 1 | 5 | 6 | E | 10735.1906 | –0.7 | –3 |
| 7 | 1 | 6 | 8 | 6 | 1 | 5 | 7 | E | 10735.2423 | 0.4 | 0 |
| 7 | 1 | 6 | 6 | 6 | 1 | 5 | 5 | E | 10735.2423 | –1.6 | –2 |
| 7 | 1 | 7 | 7 | 6 | 1 | 6 | 6 | A | 9308.7110 | –2.3 | –1 |
| 7 | 1 | 7 | 6 | 6 | 1 | 6 | 5 | A | 9308.7238 | –1.2 | 0 |
| 7 | 1 | 7 | 8 | 6 | 1 | 6 | 7 | A | 9308.7342 | 0.3 | 2 |
| 7 | 1 | 7 | 7 | 6 | 1 | 6 | 6 | E | 9308.3353 | –2.5 | –1 |
| 7 | 1 | 7 | 6 | 6 | 1 | 6 | 5 | E | 9308.3493 | –0.7 | 1 |
| 7 | 1 | 7 | 8 | 6 | 1 | 6 | 7 | E | 9308.3600 | 1.2 | 3 |
| 7 | 2 | 5 | 7 | 6 | 2 | 4 | 6 | A | 11826.4375 | –3.0 | –4 |
| 7 | 2 | 5 | 8 | 6 | 2 | 4 | 7 | A | 11826.4680 | –3.6 | –5 |
| 7 | 2 | 5 | 6 | 6 | 2 | 4 | 5 | A | 11826.4680 | –6.7 | –8 |
| 7 | 2 | 5 | 7 | 6 | 2 | 4 | 6 | E | 11747.4375 | –2.8 | –1 |

| | | | | | | | | | | | |
|---|---|---|---|---|---|---|---|---|---|---|---|
| 7 | 2 | 5 | 8 | 6 | 2 | 4 | 7 | E | 11747.4649 | –1.3 | –1 |
| 7 | 2 | 5 | 6 | 6 | 2 | 4 | 5 | E | 11747.4649 | –3.5 | –3 |
| 7 | 2 | 6 | 7 | 6 | 2 | 5 | 6 | A | 10395.1520 | 0.2 | 4 |
| 7 | 2 | 6 | 8 | 6 | 2 | 5 | 7 | A | 10395.1924 | –0.5 | 3 |
| 7 | 2 | 6 | 6 | 6 | 2 | 5 | 5 | A | 10395.1924 | –0.4 | 3 |
| 7 | 2 | 6 | 7 | 6 | 2 | 5 | 6 | E | 10421.1487 | –1.6 | 3 |
| 7 | 2 | 6 | 8 | 6 | 2 | 5 | 7 | E | 10421.1939 | –0.9 | 3 |
| 7 | 2 | 6 | 6 | 6 | 2 | 5 | 5 | E | 10421.1939 | –1.5 | 2 |
| 7 | 3 | 4 | 7 | 6 | 3 | 3 | 6 | A | 11765.1705 | –6.1 | –2 |
| 7 | 3 | 4 | 8 | 6 | 3 | 3 | 7 | A | 11765.1915 | –4.0 | 1 |
| 7 | 3 | 4 | 6 | 6 | 3 | 3 | 5 | A | 11765.1915 | –6.6 | –2 |
| 7 | 3 | 4 | 7 | 6 | 3 | 3 | 6 | E | 11541.8692 | –4.4 | –2 |
| 7 | 3 | 4 | 8 | 6 | 3 | 3 | 7 | E | 11541.8870 | –2.0 | 2 |
| 7 | 3 | 4 | 6 | 6 | 3 | 3 | 5 | E | 11541.8870 | –3.4 | 1 |
| 7 | 3 | 5 | 7 | 6 | 3 | 4 | 6 | A | 11032.9521 | –1.1 | 2 |
| 7 | 3 | 5 | 8 | 6 | 3 | 4 | 7 | A | 11033.0006 | –3.5 | 0 |
| 7 | 3 | 5 | 6 | 6 | 3 | 4 | 5 | A | 11033.0085 | –1.2 | 2 |
| 7 | 3 | 5 | 7 | 6 | 3 | 4 | 6 | E | 11264.5671 | –2.2 | 1 |
| 7 | 3 | 5 | 8 | 6 | 3 | 4 | 7 | E | 11264.6246 | –0.7 | 0 |
| 7 | 3 | 5 | 6 | 6 | 3 | 4 | 5 | E | 11264.6335 | 1.2 | 2 |
| 7 | 4 | 3 | 7 | 6 | 4 | 2 | 6 | A | 11281.4615 | –3.5 | 0 |
| 7 | 4 | 3 | 8 | 6 | 4 | 2 | 7 | A | 11281.5229 | –5.4 | –2 |
| 7 | 4 | 3 | 6 | 6 | 4 | 2 | 5 | A | 11281.5396 | 0.2 | 4 |
| 7 | 4 | 3 | 7 | 6 | 4 | 2 | 6 | E | 11203.3777 | –9.6 | –4 |
| 7 | 4 | 3 | 8 | 6 | 4 | 2 | 7 | E | 11203.4433 | –12.7 | –6 |
| 7 | 4 | 3 | 6 | 6 | 4 | 2 | 5 | E | 11203.4623 | –5.4 | 2 |
| 7 | 4 | 4 | 8 | 6 | 4 | 3 | 7 | A | 11170.1914 | –4.9 | –2 |
| 7 | 4 | 4 | 6 | 6 | 4 | 3 | 5 | A | 11170.2094 | 0.7 | 3 |
| 7 | 4 | 4 | 7 | 6 | 4 | 3 | 6 | E | 11251.1448 | 7.6 | 7 |
| 7 | 4 | 4 | 8 | 6 | 4 | 3 | 7 | E | 11251.2112 | 6.3 | 4 |
| 7 | 4 | 4 | 6 | 6 | 4 | 3 | 5 | E | 11251.2246 | 8.0 | 6 |
| 7 | 5 | 2 | 7 | 6 | 5 | 1 | 6 | A | 11124.2054 | –0.4 | 0 |
| 7 | 5 | 2 | 8 | 6 | 5 | 1 | 7 | A | 11124.3122 | –8.9 | –8 |
| 7 | 5 | 2 | 6 | 6 | 5 | 1 | 5 | A | 11124.3403 | –3.5 | –2 |
| 7 | 5 | 2 | 7 | 6 | 5 | 1 | 6 | E | 11107.3588 | –9.8 | –5 |
| 7 | 5 | 2 | 8 | 6 | 5 | 1 | 7 | E | 11107.4729 | –11.7 | –5 |
| 7 | 5 | 2 | 6 | 6 | 5 | 1 | 5 | E | 11107.4982 | –9.1 | –2 |
| 7 | 5 | 3 | 7 | 6 | 5 | 2 | 6 | A | 11118.4883 | 1.1 | 1 |
| 7 | 5 | 3 | 8 | 6 | 5 | 2 | 7 | A | 11118.5965 | –6.8 | –6 |
| 7 | 5 | 3 | 6 | 6 | 5 | 2 | 5 | A | 11118.6229 | –3.1 | –2 |
| 7 | 5 | 3 | 7 | 6 | 5 | 2 | 6 | E | 11130.4885 | 14.7 | 10 |
| 7 | 5 | 3 | 8 | 6 | 5 | 2 | 7 | E | 11130.5429 | –46.1 | 5 |
| 7 | 5 | 3 | 6 | 6 | 5 | 2 | 5 | E | 11130.5996 | –12.0 | –18 |
| 7 | 6 | 1 | 7 | 6 | 6 | 0 | 6 | A | 11069.3415 | 3.6 | 2 |
| 7 | 6 | 1 | 8 | 6 | 6 | 0 | 7 | A | 11069.5148 | 6.6 | 6 |
| 7 | 6 | 1 | 6 | 6 | 6 | 0 | 5 | A | 11069.5392 | –4.7 | –5 |
| 7 | 6 | 2 | 7 | 6 | 6 | 1 | 6 | A | 11069.2089 | –4.9 | –7 |
| 7 | 6 | 2 | 8 | 6 | 6 | 1 | 7 | A | 11069.3824 | –1.7 | –3 |

| | | | | | | | | | | | |
|---|---|---|---|---|---|---|---|---|---|---|---|
| 7 | 6 | 2 | 6 | 6 | 6 | 1 | 5 | A | 11069.4263 | 6.5 | 6 |
| 7 | 6 | 2 | 7 | 6 | 6 | 1 | 6 | E | 11072.4218 | 13.7 | 6 |
| 7 | 6 | 2 | 8 | 6 | 6 | 1 | 7 | E | 11072.5936 | 15.5 | 6 |
| 7 | 6 | 2 | 6 | 6 | 6 | 1 | 5 | E | 11072.6212 | 7.4 | –3 |
| 8 | 0 | 8 | 8 | 7 | 0 | 7 | 7 | A | 10569.9750 | –5.6 | –6 |
| 8 | 0 | 8 | 7 | 7 | 0 | 7 | 6 | A | 10569.9981 | 7.1 | 7 |
| 8 | 0 | 8 | 8 | 7 | 0 | 7 | 7 | E | 10568.0703 | –3.3 | –4 |
| 8 | 0 | 8 | 7 | 7 | 0 | 7 | 6 | E | 10568.0848 | 0.5 | 0 |
| 8 | 0 | 8 | 9 | 7 | 0 | 7 | 8 | E | 10568.0922 | 1.0 | 1 |
| 8 | 1 | 7 | 8 | 7 | 1 | 6 | 7 | A | 11933.5782 | –0.4 | 1 |
| 8 | 1 | 7 | 9 | 7 | 1 | 6 | 8 | A | 11933.6211 | –0.8 | 0 |
| 8 | 1 | 7 | 7 | 7 | 1 | 6 | 6 | A | 11933.6211 | –1.4 | 0 |
| 8 | 1 | 7 | 8 | 7 | 1 | 6 | 7 | E | 11924.8234 | –0.9 | 0 |
| 8 | 1 | 7 | 9 | 7 | 1 | 6 | 8 | E | 11924.8682 | 0.3 | 2 |
| 8 | 1 | 7 | 7 | 7 | 1 | 6 | 6 | E | 11924.8682 | –0.3 | 1 |
| 8 | 1 | 8 | 8 | 7 | 1 | 7 | 7 | A | 10558.4278 | –4.6 | –4 |
| 8 | 1 | 8 | 7 | 7 | 1 | 7 | 6 | A | 10558.4399 | –2.0 | –2 |
| 8 | 1 | 8 | 9 | 7 | 1 | 7 | 8 | A | 10558.4492 | 0.2 | 0 |
| 8 | 1 | 8 | 8 | 7 | 1 | 7 | 7 | E | 10557.3054 | –2.9 | –3 |
| 8 | 1 | 8 | 7 | 7 | 1 | 7 | 6 | E | 10557.3166 | –1.4 | –1 |
| 8 | 1 | 8 | 9 | 7 | 1 | 7 | 8 | E | 10557.3260 | 0.9 | 1 |
| 9 | 0 | 9 | 9 | 8 | 0 | 8 | 8 | A | 11809.0607 | –3.7 | –6 |
| 9 | 0 | 9 | 10 | 8 | 0 | 8 | 9 | A | 11809.0794 | 1.1 | –1 |
| 9 | 0 | 9 | 8 | 8 | 0 | 8 | 7 | A | 11809.0794 | 6.9 | 5 |
| 9 | 0 | 9 | 9 | 8 | 0 | 8 | 8 | E | 11807.2632 | –1.4 | –4 |
| 9 | 0 | 9 | 8 | 8 | 0 | 8 | 7 | E | 11807.2632 | –9.6 | –12 |
| 9 | 0 | 9 | 10 | 8 | 0 | 8 | 9 | E | 11807.2798 | 1.2 | –1 |
| 9 | 1 | 8 | 9 | 8 | 1 | 7 | 8 | A | 13121.9479 | 1.1 | 6 |
| 9 | 1 | 8 | 10 | 8 | 1 | 7 | 9 | A | 13121.9816 | –0.3 | 4 |
| 9 | 1 | 8 | 8 | 8 | 1 | 7 | 7 | A | 13121.9816 | 0.2 | 5 |
| 9 | 1 | 8 | 9 | 8 | 1 | 7 | 8 | E | 13114.6922 | –0.6 | 4 |
| 9 | 1 | 8 | 10 | 8 | 1 | 7 | 9 | E | 13114.7257 | –2.4 | 2 |
| 9 | 1 | 8 | 8 | 8 | 1 | 7 | 7 | E | 13114.7257 | –1.9 | 3 |
| 9 | 1 | 9 | 9 | 8 | 1 | 8 | 8 | A | 11804.2883 | –0.4 | –2 |
| 9 | 1 | 9 | 8 | 8 | 1 | 8 | 7 | A | 11804.2970 | 0.5 | –2 |
| 9 | 1 | 9 | 10 | 8 | 1 | 8 | 9 | A | 11804.3053 | 3.0 | 1 |
| 9 | 1 | 9 | 9 | 8 | 1 | 8 | 8 | E | 11802.8157 | 0.5 | –1 |
| 9 | 1 | 9 | 8 | 8 | 1 | 8 | 7 | E | 11802.8240 | 0.9 | –1 |
| 9 | 1 | 9 | 10 | 8 | 1 | 8 | 9 | E | 11802.8324 | 3.4 | 2 |
| 10 | 0 | 10 | 10 | 9 | 0 | 9 | 9 | A | 13050.3358 | 1.1 | –4 |
| 10 | 0 | 10 | 9 | 9 | 0 | 9 | 8 | A | 13050.3445 | 3.2 | –2 |
| 10 | 0 | 10 | 11 | 9 | 0 | 9 | 10 | A | 13050.3504 | 4.2 | –1 |
| 10 | 0 | 10 | 10 | 9 | 0 | 9 | 9 | E | 13048.5670 | 9.1 | 4 |
| 10 | 0 | 10 | 9 | 9 | 0 | 9 | 8 | E | 13048.5755 | 11.0 | 6 |
| 10 | 0 | 10 | 11 | 9 | 0 | 9 | 10 | E | 13048.5806 | 11.2 | 6 |

**Table S6.** Rotational constants of 4MNP-1 calculated at different levels of theory. The difference between the calculated and the experimental (in MHz) are given as *ΔA*, *ΔB* and *ΔC*. *Σ* is the sum of the absolute values of *ΔA*, *ΔB* and *ΔC*.

| Method/Basis set | *A* | Δ*A* | *B* | Δ*B* | *C* | Δ*C* | Σ |
|---|---|---|---|---|---|---|---|
| B3LYP-D3/6-31G(d,p) | 1843.5 | 1.8 | 928.1 | –4.0 | 619.7 | –1.9 | 7.6 |
| B3LYP-D3/6-31+G(d,p) | 1837.8 | –4.0 | 926.7 | –5.4 | 618.4 | –3.1 | 12.5 |
| B3LYP-D3/6-31++G(d,p) | 1837.8 | –4.0 | 926.7 | –5.4 | 618.4 | –3.2 | 12.5 |
| B3LYP-D3/6-311G(d,p) | 1844.2 | 2.5 | 930.0 | –2.1 | 620.6 | –0.9 | 5.5 |
| B3LYP-D3/6-311+G(d,p) | 1841.9 | 0.1 | 929.7 | –2.3 | 620.2 | –1.3 | 3.8 |
| B3LYP-D3/6-311++G(d,p) | 1841.9 | 0.2 | 929.7 | –2.3 | 620.2 | –1.3 | 3.8 |
| B3LYP-D3/6-311G(2d,2p) | 1852.8 | 11.1 | 932.4 | 0.4 | 622.7 | 1.1 | 12.6 |
| B3LYP-D3/6-311+G(2d,2p) | 1850.3 | 8.5 | 932.4 | 0.3 | 622.4 | 0.8 | 9.7 |
| B3LYP-D3/6-311++G(2d,2p) | 1850.3 | 8.6 | 932.4 | 0.3 | 622.4 | 0.8 | 9.7 |
| B3LYP-D3/6-311G(df,pd) | 1851.5 | 9.7 | 932.2 | 0.2 | 622.4 | 0.9 | 10.8 |
| B3LYP-D3/6-311+G(df,pd) | 1849.0 | 7.3 | 932.1 | 0.0 | 622.1 | 0.5 | 7.8 |
| B3LYP-D3/6-311++G(df,pd) | 1849.1 | 7.4 | 932.1 | 0.0 | 622.1 | 0.5 | 7.9 |
| B3LYP-D3/6-311G(2df,2pd) | 1856.8 | 15.1 | 933.6 | 1.5 | 623.6 | 2.1 | 18.7 |
| B3LYP-D3/6-311+G(2df,2pd) | 1854.6 | 12.9 | 933.4 | 1.3 | 623.3 | 1.7 | 15.9 |
| B3LYP-D3/6-311++G(2df,2pd) | 1854.7 | 12.9 | 933.4 | 1.3 | 623.3 | 1.7 | 15.9 |
| B3LYP-D3/6-311G(3df,3pd) | 1857.3 | 15.5 | 933.6 | 1.6 | 623.7 | 2.1 | 19.2 |
| B3LYP-D3/6-311+G(3df,3pd) | 1855.5 | 13.7 | 933.7 | 1.7 | 623.5 | 2.0 | 17.4 |
| B3LYP-D3/6-311++G(3df,3pd) | 1855.5 | 13.8 | 933.8 | 1.7 | 623.6 | 2.0 | 17.5 |
| B3LYP-D3/cc-pVDZ | 1844.4 | 2.6 | 925.2 | –6.8 | 618.5 | –3.0 | 12.5 |
| B3LYP-D3/aug-cc-pVDZ | 1840.0 | 1.8 | 925.5 | –6.6 | 618.1 | –3.5 | 11.9 |
| B3LYP-D3/cc-pVTZ | 1855.4 | 13.6 | 933.0 | 0.9 | 623.2 | 1.6 | 16.2 |
| B3LYP-D3/aug-cc-pVTZ | 1854.5 | 12.7 | 933.3 | 1.2 | 623.2 | 1.6 | 15.5 |
| | | | | | | | |
| B3LYP-D3BJ/6-31G(d,p) | 1847.8 | 6.0 | 930.4 | –1.6 | 621.2 | –0.4 | 8.0 |
| B3LYP-D3BJ/6-31+G(d,p) | 1842.3 | 0.6 | 929.1 | –3.0 | 620.0 | –1.6 | 5.1 |
| B3LYP-D3BJ/6-31++G(d,p) | 1842.3 | 0.5 | 929.1 | –3.0 | 620.0 | –1.6 | 5.1 |
| B3LYP-D3BJ/6-311G(d,p) | 1848.5 | 6.7 | 932.4 | 0.4 | 622.2 | 0.6 | 7.7 |
| B3LYP-D3BJ/6-311+G(d,p) | 1846.2 | 4.5 | 932.2 | 0.2 | 621.8 | 0.3 | 4.9 |
| B3LYP-D3BJ/6-311++G(d,p) | 1846.2 | 4.5 | 932.2 | 0.1 | 621.8 | 0.3 | 4.9 |
| B3LYP-D3BJ/6-311G(2d,2p) | 1857.0 | 15.3 | 934.9 | 2.9 | 624.2 | 2.7 | 20.8 |
| B3LYP-D3BJ/6-311+G(2d,2p) | 1854.7 | 12.9 | 934.8 | 2.8 | 623.9 | 2.4 | 18.0 |
| B3LYP-D3BJ/6-311++G(2d,2p) | 1854.7 | 12.9 | 934.8 | 2.8 | 623.9 | 2.4 | 18.1 |
| B3LYP-D3BJ/6-311G(df,pd) | 1855.8 | 14.0 | 934.7 | 2.6 | 624.0 | 2.4 | 19.1 |
| B3LYP-D3BJ/6-311+G(df,pd) | 1853.5 | 11.8 | 934.5 | 2.5 | 623.7 | 2.1 | 16.3 |
| B3LYP-D3BJ/6-311++G(df,pd) | 1853.6 | 11.8 | 934.5 | 2.4 | 623.7 | 2.1 | 16.4 |
| B3LYP-D3BJ/6-311G(2df,2pd) | 1857.0 | 15.3 | 934.9 | 2.9 | 624.2 | 2.7 | 20.8 |
| B3LYP-D3BJ/6-311+G(2df,2pd) | 1854.7 | 12.9 | 934.8 | 2.8 | 623.9 | 2.4 | 18.0 |
| B3LYP-D3BJ/6-311++G(2df,2pd) | 1854.7 | 12.9 | 934.8 | 2.8 | 623.9 | 2.4 | 18.1 |
| B3LYP-D3BJ/6-311G(3df,3pd) | 1861.5 | 19.7 | 936.1 | 4.1 | 625.3 | 3.7 | 27.5 |
| B3LYP-D3BJ/6-311+G(3df,3pd) | 1859.8 | 18.1 | 936.2 | 4.2 | 625.2 | 3.6 | 25.8 |
| B3LYP-D3BJ/6-311++G(3df,3pd) | 1859.8 | 18.0 | 936.2 | 4.2 | 625.1 | 3.6 | 25.8 |
| B3LYP-D3BJ/cc-pVDZ | 1848.4 | 6.6 | 927.7 | –4.4 | 620.1 | –1.5 | 12.5 |
| B3LYP-D3BJ/aug-cc-pVDZ | 1844.3 | 2.5 | 927.9 | –4.1 | 619.7 | –1.8 | 8.5 |
| B3LYP-D3BJ/cc-pVTZ | 1859.5 | 17.8 | 935.5 | 3.4 | 624.8 | 3.2 | 24.4 |

| | | | | | | | |
|---|---|---|---|---|---|---|---|
| B3LYP-D3BJ/aug-cc-pVTZ | 1858.7 | 17.0 | 935.8 | 3.7 | 624.8 | 3.3 | 24.0 |
| | | | | | | | |
| CAM-B3LYP-D3BJ/6-311G(d,p) | 1862.3 | 20.5 | 939.5 | 7.5 | 626.9 | 5.3 | 33.3 |
| CAM-B3LYP-D3BJ/6-311+G(d,p) | 1859.8 | 18.0 | 939.3 | 7.2 | 626.5 | 4.9 | 30.2 |
| CAM-B3LYP-D3BJ/6-311++G(d,p) | 1859.8 | 18.0 | 939.3 | 7.2 | 626.5 | 5.0 | 30.2 |
| CAM-B3LYP-D3BJ/cc-pVDZ | 1861.9 | 20.1 | 934.9 | 2.9 | 624.8 | 3.3 | 26.3 |
| CAM-B3LYP-D3BJ/aug-cc-pVDZ | 1857.5 | 15.7 | 935.0 | 2.9 | 624.4 | 2.8 | 21.5 |
| CAM-B3LYP-D3BJ/cc-pVTZ | 1873.1 | 31.3 | 942.6 | 10.6 | 629.5 | 7.9 | 49.8 |
| CAM-B3LYP-D3BJ/aug-cc-pVTZ | 1872.1 | 30.3 | 942.9 | 10.8 | 629.5 | 7.9 | 49.1 |
| | | | | | | | |
| CCSD/cc-pVDZ | 1823.7 | –18.0 | 921.9 | –10.1 | 614.8 | –6.8 | 35.0 |
| | | | | | | | |
| M06-2X/6-31G(d,p) | 1849.1 | 7.3 | 936.8 | 4.7 | 624.2 | 2.6 | 14.7 |
| M06-2X/6-31+G(d,p) | 1843.6 | 1.9 | 935.6 | 3.6 | 623.1 | 1.5 | 7.0 |
| M06-2X/6-31++G(d,p) | 1843.6 | 1.9 | 935.6 | 3.5 | 623.0 | 1.5 | 6.9 |
| M06-2X/6-311G(d,p) | 1850.2 | 8.5 | 938.0 | 5.9 | 624.8 | 3.3 | 17.6 |
| M06-2X/6-311+G(d,p) | 1848.2 | 6.4 | 937.7 | 5.6 | 624.5 | 2.9 | 14.9 |
| M06-2X/6-311++G(d,p) | 1848.1 | 6.4 | 937.7 | 5.6 | 624.5 | 2.9 | 15.0 |
| M06-2X/6-311G(2d,2p) | 1858.3 | 16.6 | 940.1 | 8.1 | 626.7 | 5.1 | 29.8 |
| M06-2X/6-311+G(2d,2p) | 1855.9 | 14.2 | 940.0 | 7.9 | 626.4 | 4.8 | 26.9 |
| M06-2X/6-311++G(2d,2p) | 1856.0 | 14.2 | 940.0 | 7.9 | 626.4 | 4.8 | 27.0 |
| M06-2X/6-311G(df,pd) | 1857.5 | 15.7 | 939.4 | 7.3 | 626.3 | 4.7 | 27.8 |
| M06-2X/6-311+G(df,pd) | 1854.9 | 13.1 | 939.2 | 7.2 | 625.9 | 4.4 | 24.7 |
| M06-2X/6-311++G(df,pd) | 1854.9 | 13.1 | 939.3 | 7.2 | 625.9 | 4.4 | 24.7 |
| M06-2X/6-311G(2df,2pd) | 1863.6 | 21.8 | 940.6 | 8.6 | 627.5 | 6.0 | 36.4 |
| M06-2X/6-311+G(2df,2pd) | 1861.2 | 19.4 | 940.4 | 8.4 | 627.2 | 5.6 | 33.4 |
| M06-2X/6-311++G(2df,2pd) | 1861.2 | 19.4 | 940.4 | 8.4 | 627.2 | 5.6 | 33.4 |
| M06-2X/6-311G(3df,3pd) | 1863.4 | 21.6 | 940.5 | 8.4 | 627.4 | 5.9 | 35.9 |
| M06-2X/6-311+G(3df,3pd) | 1861.6 | 19.8 | 940.6 | 8.6 | 627.3 | 5.7 | 34.1 |
| M06-2X/6-311++G(3df,3pd) | 1861.6 | 19.8 | 940.6 | 8.6 | 627.3 | 5.7 | 34.1 |
| M06-2X/cc-pVDZ | 1851.7 | 9.9 | 934.6 | 2.5 | 623.5 | 2.0 | 14.4 |
| M06-2X/aug-cc-pVDZ | 1847.8 | 6.0 | 934.2 | 2.1 | 622.9 | 1.3 | 9.4 |
| M06-2X/cc-pVTZ | 1862.3 | 20.5 | 940.3 | 8.2 | 627.2 | 5.7 | 34.4 |
| M06-2X/aug-cc-pVTZ | 1861.0 | 19.2 | 940.6 | 8.5 | 627.2 | 5.7 | 33.4 |
| | | | | | | | |
| MN15/6-31G(d,p) | 1845.2 | 3.5 | 934.2 | 2.1 | 622.6 | 1.0 | 6.6 |
| MN15/6-31+G(d,p) | 1839.5 | –2.2 | 932.7 | 0.6 | 621.3 | –0.3 | 3.1 |
| MN15/6-31++G(d,p) | 1839.5 | –2.2 | 932.7 | 0.6 | 621.3 | –0.3 | 3.1 |
| MN15/6-311G(d,p) | 1849.0 | 7.2 | 937.0 | 5.0 | 624.3 | 2.7 | 14.9 |
| MN15/6-311+G(d,p) | 1846.8 | 5.1 | 936.7 | 4.7 | 623.9 | 2.3 | 12.0 |
| MN15/6-311++G(d,p) | 1846.8 | 5.0 | 936.8 | 4.7 | 623.9 | 2.4 | 12.1 |
| MN15/6-311G(2d,2p) | 1857.4 | 15.6 | 939.5 | 7.4 | 626.3 | 4.8 | 27.8 |
| MN15/6-311+G(2d,2p) | 1855.1 | 13.4 | 939.3 | 7.2 | 626.0 | 4.4 | 25.0 |
| MN15/6-311++G(2d,2p) | 1855.2 | 13.4 | 939.3 | 7.2 | 626.0 | 4.4 | 25.0 |
| MN15/6-311G(df,pd) | 1855.6 | 13.8 | 939.8 | 7.7 | 626.2 | 4.7 | 26.2 |
| MN15/6-311+G(df,pd) | 1853.2 | 11.5 | 939.4 | 7.4 | 625.8 | 4.3 | 23.1 |
| MN15/6-311++G(df,pd) | 1853.2 | 11.4 | 939.5 | 7.4 | 625.8 | 4.3 | 23.1 |
| MN15/6-311G(2df,2pd) | 1860.8 | 19.0 | 940.6 | 8.6 | 627.2 | 5.6 | 33.2 |
| MN15/6-311+G(2df,2pd) | 1858.8 | 17.1 | 940.3 | 8.2 | 626.8 | 5.3 | 30.6 |
| MN15/6-311++G(2df,2pd) | 1858.8 | 17.1 | 940.3 | 8.2 | 626.8 | 5.3 | 30.6 |

| | | | | | | | |
|---|---|---|---|---|---|---|---|
| MN15/6-311G(3df,3pd) | 1861.8 | 20.1 | 941.0 | 8.9 | 627.5 | 5.9 | 34.9 |
| MN15/6-311+G(3df,3pd) | 1860.4 | 18.7 | 941.0 | 8.9 | 627.3 | 5.8 | 33.4 |
| MN15/6-311++G(3df,3pd) | 1860.5 | 18.7 | 941.0 | 8.9 | 627.3 | 5.8 | 33.4 |
| MN15/cc-pVDZ | 1849.3 | 7.6 | 932.3 | 0.2 | 622.2 | 0.7 | 8.4 |
| MN15/aug-cc-pVDZ | 1845.2 | 3.4 | 932.7 | 0.7 | 622.0 | 0.4 | 4.5 |
| MN15/cc-pVTZ | 1860.5 | 18.7 | 940.4 | 8.4 | 627.1 | 5.5 | 32.6 |
| MN15/aug-cc-pVTZ | 1860.5 | 18.7 | 941.1 | 9.0 | 627.4 | 5.8 | 33.5 |
| | | | | | | | |
| PBE0/6-31G(d,p) | 1837.9 | –3.8 | 920.3 | –11.8 | 615.6 | –6.0 | 21.6 |
| PBE0/6-31+G(d,p) | 1833.0 | –8.7 | 918.8 | –13.2 | 614.4 | –7.2 | 29.1 |
| PBE0/6-31++G(d,p) | 1833.0 | –8.8 | 918.8 | –13.2 | 614.4 | –7.2 | 29.1 |
| PBE0/6-311G(d,p) | 1838.3 | –3.4 | 922.6 | –9.4 | 616.7 | –4.9 | 17.7 |
| PBE0/6-311+G(d,p) | 1836.1 | –5.6 | 922.3 | –9.7 | 616.3 | –5.2 | 20.6 |
| PBE0/6-311++G(d,p) | 1836.2 | –5.6 | 922.4 | –9.7 | 616.3 | –5.2 | 20.5 |
| PBE0/6-311G(2d,2p) | 1846.9 | 5.1 | 924.6 | –7.5 | 618.5 | –3.0 | 15.6 |
| PBE0/6-311+G(2d,2p) | 1844.6 | 2.8 | 924.6 | –7.5 | 618.2 | –3.3 | 13.6 |
| PBE0/6-311++G(2d,2p) | 1844.6 | 2.9 | 924.5 | –7.5 | 618.2 | –3.3 | 13.7 |
| PBE0/6-311G(df,pd) | 1845.5 | 3.8 | 924.7 | –7.4 | 618.4 | –3.1 | 14.3 |
| PBE0/6-311+G(df,pd) | 1843.5 | 1.7 | 924.4 | –7.7 | 618.1 | –3.5 | 12.9 |
| PBE0/6-311++G(df,pd) | 1843.5 | 1.8 | 924.4 | –7.7 | 618.1 | –3.5 | 12.9 |
| PBE0/6-311G(2df,2pd) | 1850.5 | 8.7 | 925.8 | –6.3 | 619.4 | –2.1 | 17.1 |
| PBE0/6-311+G(2df,2pd) | 1848.7 | 6.9 | 925.5 | –6.5 | 619.1 | –2.4 | 15.9 |
| PBE0/6-311++G(2df,2pd) | 1848.7 | 6.9 | 925.5 | –6.5 | 619.1 | –2.4 | 15.9 |
| PBE0/6-311G(3df,3pd) | 1851.2 | 9.5 | 925.9 | –6.2 | 619.6 | –2.0 | 17.6 |
| PBE0/6-311+G(3df,3pd) | 1849.6 | 7.8 | 926.0 | –6.1 | 619.4 | –2.1 | 16.0 |
| PBE0/6-311++G(3df,3pd) | 1849.6 | 7.8 | 926.0 | –6.1 | 619.4 | –2.1 | 16.0 |
| PBE0/cc-pVDZ | 1839.3 | –2.4 | 918.1 | –14.0 | 614.8 | –6.8 | 23.2 |
| PBE0/aug-cc-pVDZ | 1834.5 | –7.3 | 918.3 | –13.7 | 614.4 | –7.2 | 28.2 |
| PBE0/cc-pVTZ | 1849.2 | 7.4 | 925.1 | –6.9 | 619.0 | –2.5 | 16.9 |
| PBE0/aug-cc-pVTZ | 1848.3 | 6.5 | 925.4 | –6.7 | 619.0 | –2.5 | 15.7 |
| | | | | | | | |
| PBE0-D3/6-31G(d,p) | 1837.4 | –4.3 | 920.1 | –12.0 | 615.5 | –6.1 | 22.4 |
| PBE0-D3/6-31+G(d,p) | 1832.4 | –9.4 | 918.7 | –13.3 | 614.3 | –7.3 | 30.0 |
| PBE0-D3/6-31++G(d,p) | 1832.4 | –9.4 | 918.7 | –13.3 | 614.3 | –7.3 | 30.0 |
| PBE0-D3/6-311G(d,p) | 1837.8 | –4.0 | 922.5 | –9.6 | 616.6 | –5.0 | 18.6 |
| PBE0-D3/6-311+G(d,p) | 1835.6 | –6.2 | 922.2 | –9.9 | 616.2 | –5.4 | 21.4 |
| PBE0-D3/6-311++G(d,p) | 1835.6 | –6.2 | 922.2 | –9.9 | 616.2 | –5.4 | 21.4 |
| PBE0-D3/6-311G(2d,2p) | 1846.2 | 4.5 | 924.5 | –7.6 | 618.4 | –3.2 | 15.2 |
| PBE0-D3/6-311+G(2d,2p) | 1844.0 | 2.3 | 924.4 | –7.7 | 618.1 | –3.5 | 13.4 |
| PBE0-D3/6-311++G(2d,2p) | 1844.1 | 2.3 | 924.4 | –7.7 | 618.1 | –3.4 | 13.4 |
| PBE0-D3/6-311G(df,pd) | 1845.0 | 3.2 | 924.5 | –7.5 | 618.3 | –3.3 | 14.1 |
| PBE0-D3/6-311+G(df,pd) | 1842.9 | 1.2 | 924.2 | –7.9 | 617.9 | –3.6 | 12.7 |
| PBE0-D3/6-311++G(df,pd) | 1842.9 | 1.2 | 924.2 | –7.8 | 617.9 | –3.6 | 12.7 |
| PBE0-D3/6-311G(2df,2pd) | 1850.0 | 8.2 | 925.6 | –6.5 | 619.3 | –2.2 | 17.0 |
| PBE0-D3/6-311+G(2df,2pd) | 1848.2 | 6.4 | 925.4 | –6.7 | 619.0 | –2.6 | 15.7 |
| PBE0-D3/6-311++G(2df,2pd) | 1848.2 | 6.4 | 925.4 | –6.7 | 619.0 | –2.5 | 15.7 |
| PBE0-D3/6-311G(3df,3pd) | 1850.8 | 9.0 | 925.7 | –6.4 | 619.4 | –2.1 | 17.5 |
| PBE0-D3/6-311+G(3df,3pd) | 1849.1 | 7.3 | 925.8 | –6.3 | 619.3 | –2.3 | 15.9 |
| PBE0-D3/6-311++G(3df,3pd) | 1849.1 | 7.3 | 925.8 | –6.3 | 619.3 | –2.3 | 15.9 |
| PBE0-D3/cc-pVDZ | 1838.9 | –2.8 | 917.9 | –14.2 | 614.7 | –6.9 | 23.9 |

| | | | | | | | |
|---|---|---|---|---|---|---|---|
| PBE0-D3/aug-cc-pVDZ | 1834.0 | –7.8 | 918.2 | –13.9 | 614.2 | –7.3 | 29.0 |
| PBE0-D3/cc-pVTZ | 1848.8 | 7.0 | 925.0 | –7.1 | 618.9 | –2.7 | 16.8 |
| PBE0-D3/aug-cc-pVTZ | 1847.8 | 6.0 | 925.2 | –6.9 | 618.9 | –2.7 | 15.5 |
| | | | | | | | |
| ωB97X-D/6-31G(d,p) | 1852.7 | 11.0 | 935.7 | 3.6 | 624.1 | 2.5 | 17.1 |
| ωB97X-D/6-31+G(d,p) | 1848.1 | 6.3 | 934.7 | 2.6 | 623.1 | 1.6 | 10.6 |
| ωB97X-D/6-31++G(d,p) | 1848.1 | 6.3 | 934.7 | 2.6 | 623.1 | 1.6 | 10.5 |
| ωB97X-D-6-311G(d,p) | 1854.5 | 12.7 | 937.6 | 5.6 | 625.2 | 3.6 | 21.9 |
| ωB97X-D-6-311+G(d,p) | 1852.5 | 10.7 | 937.5 | 5.5 | 624.9 | 3.4 | 19.6 |
| ωB97X-D-6-311++G(d,p) | 1852.5 | 10.8 | 937.5 | 5.5 | 624.9 | 3.3 | 19.6 |
| ωB97X-D/6-311G(2d,2p) | 1862.7 | 21.0 | 939.9 | 7.8 | 627.1 | 5.5 | 34.3 |
| ωB97X-D/6-311+G(2d,2p) | 1860.7 | 18.9 | 939.9 | 7.8 | 626.9 | 5.3 | 32.0 |
| ωB97X-D/6-311++G(2d,2p) | 1860.7 | 18.9 | 939.8 | 7.8 | 626.9 | 5.3 | 32.0 |
| ωB97X-D/6-311G(df,pd) | 1860.7 | 18.9 | 939.5 | 7.5 | 626.7 | 5.2 | 31.6 |
| ωB97X-D/6-311+G(df,pd) | 1858.7 | 16.9 | 939.4 | 7.4 | 626.5 | 4.9 | 29.2 |
| ωB97X-D/6-311++G(df,pd) | 1858.7 | 16.9 | 939.5 | 7.4 | 626.5 | 4.9 | 29.2 |
| ωB97X-D/6-311G(2df,2pd) | 1866.0 | 24.3 | 940.8 | 8.8 | 627.9 | 6.3 | 39.4 |
| ωB97X-D/6-311+G(2df,2pd) | 1864.3 | 22.5 | 940.7 | 8.6 | 627.6 | 6.1 | 37.2 |
| ωB97X-D/6-311++G(2df,2pd) | 1864.4 | 22.6 | 940.6 | 8.6 | 627.6 | 6.1 | 37.2 |
| ωB97X-D/6-311G(3df,3pd) | 1866.3 | 24.5 | 940.8 | 8.8 | 627.9 | 6.4 | 39.7 |
| ωB97X-D/6-311+G(3df,3pd) | 1864.9 | 23.1 | 941.0 | 8.9 | 627.8 | 6.3 | 38.3 |
| ωB97X-D/6-311++G(3df,3pd) | 1864.9 | 23.2 | 940.9 | 8.9 | 627.8 | 6.3 | 38.3 |
| ωB97X-D/cc-pVDZ | 1853.1 | 11.4 | 933.2 | 1.2 | 623.1 | 1.5 | 14.0 |
| ωB97X-D/aug-cc-pVDZ | 1849.8 | 8.0 | 933.1 | 1.1 | 622.7 | 1.1 | 10.2 |
| ωB97X-D/cc-pVTZ | 1864.8 | 23.0 | 940.7 | 8.6 | 627.7 | 6.1 | 37.8 |
| ωB97X-D/aug-cc-pVTZ | 1865.5 | 23.7 | 940.4 | 8.3 | 627.6 | 6.1 | 38.1 |
| | | | | | | | |
| MP2/6-31G(d,p) | 1826.4 | –15.4 | 931.4 | –0.7 | 619.2 | –2.4 | 18.4 |
| MP2/6-31+G(d,p) | 1819.1 | –22.7 | 929.9 | –2.1 | 617.8 | –3.8 | 28.6 |
| MP2/6-31++G(d,p) | 1818.8 | –23.0 | 930.0 | –2.0 | 617.8 | –3.8 | 28.8 |
| MP2/6-311G(d,p) | 1830.9 | –10.9 | 929.2 | –2.8 | 618.8 | –2.8 | 16.5 |
| MP2/6-311+G(d,p) | 1827.9 | –13.9 | 927.8 | –4.3 | 619.3 | –2.3 | 20.5 |
| MP2/6-311++G(d,p) | 1827.8 | –14.0 | 928.0 | –4.0 | 619.0 | –2.6 | 20.6 |
| MP2/6-311G(2d,2p) | 1837.4 | –4.4 | 934.0 | 2.0 | 621.6 | 0.0 | 6.4 |
| MP2/6-311+G(2d,2p) | 1834.8 | –6.9 | 933.5 | 1.5 | 621.1 | –0.5 | 8.9 |
| MP2/6-311++G(2d,2p) | 1834.8 | –7.0 | 933.5 | 1.4 | 621.1 | –0.5 | 8.9 |
| MP2/6-311G(df,pd) | 1853.6 | 11.8 | 934.5 | 2.4 | 623.7 | 2.1 | 16.4 |
| MP2/6-311+G(df,pd) | 1850.5 | 8.7 | 933.0 | 0.9 | 624.1 | 2.5 | 12.1 |
| MP2/6-311++G(df,pd) | 1850.5 | 8.7 | 933.1 | 1.0 | 623.8 | 2.3 | 12.0 |
| MP2/6-311G(2df,2pd) | 1852.5 | 10.7 | 938.7 | 6.6 | 625.4 | 3.9 | 21.2 |
| MP2/6-311+G(2df,2pd) | 1849.7 | 7.9 | 937.8 | 5.7 | 624.7 | 3.1 | 16.7 |
| MP2/6-311++G(2df,2pd) | 1849.7 | 7.9 | 937.8 | 5.7 | 624.7 | 3.1 | 16.7 |
| MP2/6-311G(3df,3pd) | 1849.6 | 7.8 | 937.1 | 5.0 | 624.4 | 2.8 | 15.6 |
| MP2/6-311+G(3df,3pd) | 1847.3 | 5.6 | 936.9 | 4.8 | 624.0 | 2.4 | 12.8 |
| MP2/6-311++G(3df,3pd) | 1847.3 | 5.6 | 936.9 | 4.8 | 624.0 | 2.4 | 12.8 |
| MP2/cc-pVDZ | 1821.4 | –20.3 | 922.3 | –9.8 | 614.6 | –6.9 | 37.0 |
| MP2/aug-cc-pVDZ | 1814.1 | –27.7 | 920.8 | –11.3 | 613.1 | –8.4 | 47.4 |
| MP2/cc-pVTZ | 1847.9 | 6.2 | 936.3 | 4.2 | 623.8 | 2.3 | 12.7 |
| MP2/aug-cc-pVTZ | 1844.0 | 2.2 | 936.4 | 4.3 | 623.4 | 1.8 | 8.3 |
| **Experimental** | **1841.8** | | **932.1** | | **621.6** | | |
